# Plasma vitamin C levels are associated with brain structural networks on MRI: A large cohort study

**DOI:** 10.1371/journal.pone.0348504

**Published:** 2026-06-10

**Authors:** Haruka Nagaya, Keita Watanabe, Tomohiro Shintaku, Miho Sasaki, Jusei Kudo, Sera Kasai, Yuka Ishimoto, Kana Saito, Shuichi Matsuhashi, Taiki Koshiishi, Mizuki Imura, Amo Ozawa, Saaya Mori, Daisuke Watanabe, Shin Shukunobe, Tatsuro Sasaki, Soichiro Tatsuo, Shinya Kakehata, Tatsuya Mikami, Daichi Kokubu, Yusuke Ushida, Shingo Kakeda

**Affiliations:** 1 Department of Radiology, Graduate School of Medicine, Hirosaki University, Hirosaki, Japan; 2 Department of Radiology, Kyoto Prefectural University of Medicine, Kyoto, Japan; 3 Innovation Center for Health Promotion, Hirosaki University, Hirosaki, Japan; 4 Diet & Well-being Research Institute, KAGOME CO., LTD., Nasushiobara, Japan; Nagoya University: Nagoya Daigaku, JAPAN

## Abstract

**Background:**

Neurodegenerative diseases significantly impact brain health in older adults, and although dietary vitamin C intake has been associated with a reduced risk of cognitive impairment, it remains unclear whether plasma vitamin C levels independently affect brain structure and neural connectivity. This study aimed to investigate whether plasma vitamin C levels were independently associated with brain volume and default mode network (DMN) connectivity in older adults.

**Methods:**

All participants underwent 3T magnetic resonance imaging (MRI). Total intracranial volume (ICV), gray matter volume (GMV), and white matter volume (WMV) were calculated using CAT 12 in SPM 12. DMN connectivity was assessed using independent component analysis, based on shared GMV variance across voxels, and quantified by the loading coefficients. Multiple regression analysis was used to investigate the associations among brain volume, DMN connectivity measurements, and plasma vitamin C levels. These analyses were adjusted for potential confounders (age, sex, Mini-Mental State Examination score, diabetes, hypertension, hyperlipidemia, and education levels), and lifestyle factors (smoking history, drinking history, and physical activity). GMV/ICV ratio and WMV/ICV ratio were calculated to adjust for individual differences in head size.

**Results:**

This cross-sectional study included 2,044 participants (median age, 69 years; females, 61.1%). Low plasma vitamin C levels were significantly and independently associated with the GMV/ICV ratio (p < 0.001) and DMN connectivity (p < 0.001).

**Conclusions:**

In conclusion, our findings demonstrate that plasma vitamin C levels are positively associated with the structural integrity of the gray matter and DMN connectivity, generating the hypothesis that vitamin C may play a role in brain health.

## Introduction

Vitamin C is essential for improving brain health. It is a crucial antioxidant in the brain that plays a role in several biological functions, including acting as a cofactor in various enzymatic reactions, serving as a neuromodulator, and being involved in regulating certain peptide hormones. Human studies have reported that vitamin C concentrations are more than 2-fold higher in the cerebrospinal fluid (micromolar range) compared to plasma levels [1,2]. In experimental study, vitamin C concentrations in mice neurons reached 10,000 μM [3]. The role of vitamin C in promoting brain function has been explored in previous in vivo and ex vivo studies. A study on genetically modified mice showed that long-term high vitamin C intake was important for maintaining brain cholesterol homeostasis and preventing age-related oxidative damage [4] Dietary antioxidants significantly contribute to the protective effect against oxidative damage and the maintenance of neuronal function [5].

A previous study provided evidence of the association between vitamin C intake and the development of dementia. Engelhart et al. [6] studied the association between vitamin C intake and the development of Alzheimer’s Disease (AD) using 5,395 male and female participants from the Rotterdam Scan Study. They reported that higher vitamin C intake was associated with an 18% lower relative risk (RR) for AD (RR: 0.82, 95% confidence interval (CI): 0.68–0.99). As such, its potential therapeutic applications in neurodegenerative diseases such as AD, Parkinson’s disease (PD), Huntington’s disease, multiple sclerosis, and amyotrophic lateral sclerosis, as well as in psychiatric disorders including depression, anxiety, and schizophrenia, are being investigated [7–12]. Although investigating the association between plasma vitamin C levels and brain magnetic resonance imaging (MRI) measurements may identify markers of neuronal integrity that may help prevent cognitive decline, few studies have focused on the effects of plasma vitamin C levels on brain MRI measurements. To the best of our knowledge, only one previous study demonstrated that decreased gray matter (GM) volume (GMV) was associated with reduced plasma vitamin C levels in older adults [13]. Furthermore, no studies have evaluated the association between plasma vitamin C levels and brain networks.

The default mode network (DMN) is a collection of brain regions with highly correlated activity, including the ventromedial prefrontal cortex and posterior cingulate cortex (PCC), along with the precuneus, inferior parietal cortex, and lateral temporal cortices [14]. These brain regions are active during resting or task-independent cognitive states. The DMN is associated with various cognitive functions, including autobiographical memory, future thinking, self-reference, attention, and theory of mind [15]. Recently, source-based morphometry (SBM) has emerged as a multivariate analytical approach for three-dimensional (3D) T1-weighted structural MRI. While functional MRI (fMRI) is predominantly confined to research settings and less applicable in routine clinical practice, SBM provides a practical alternative for investigating structural brain networks, making it highly suitable for large-scale epidemiological imaging studies. SBM is a multivariate, data-driven method that applies independent component analysis (ICA) to structural MRI data to extract spatially independent GM structural networks based on shared variance across voxels [16]. Unlike voxel-based morphometry (VBM), which focuses on voxel-wise group comparisons, SBM identifies naturally occurring patterns of covariance among brain regions. Therefore, SBM may provide insights into the structural connectivity or network-level alterations underlying various neurological and psychiatric conditions.

This study aimed to evaluate the association between plasma vitamin C levels and DMN connectivity, as well as global and regional brain volume (using VBM) by using data from a population-based prospective study. We hypothesized that plasma vitamin C levels were associated with DMN connectivity and preserved brain volume.

## Methods

### Ethics statement

This study was approved by the Ethics Committee of Hirosaki University Graduate School of Medicine. It was initially approved on May 25, 2016 (reference number: 2016−029), and subsequently updated and re-approved under the current authorization number 2019−064 (most recently approved as 2019-064-5 on March 6, 2025). The study was conducted in accordance with the ethical guidelines of the Declaration of Helsinki. For research purposes, the authors accessed the de-identified data from 01/01/2023 to 31/12/2023. Written informed consent was obtained from all participants.

### Study population and study design

This cross-sectional study used data from The Iki-Iki Health Promotion Project. Briefly, the project was established in 2016 as a population-based prospective study of cerebrovascular and cardiovascular diseases and dementia in the older Japanese population from the Iwaki area of Hirosaki City, located in western Aomori Prefecture, Japan. Overall, residents aged >64 years participated in the screening surveys in 2016 and 2017.

### Blood sampling and testing

Blood samples were collected from the median cubital vein after an overnight fast. Blood vitamin C levels were measured by KAGOME CO., LTD. The plasma concentration of vitamin C (ascorbic acid) was measured using a commercially available kit (R01K02; Shima Laboratories Co. Ltd., Tokyo, Japan).

### MRI acquisition

All brain MRI data were obtained using the same protocol on a single 3T MRI scanner (Signa EXCITE 3T; GE Healthcare, Waukesha, WI, USA) with an 8-channel brain phased-array coil. Original T1-weighted images were acquired in the steady state using a 3D fast-spoiled gradient echo sequence with the following parameters: repetition time, 10 ms; echo time, 4.1 ms; inversion time, 700 ms; flip angle, 10 degrees; field-of-view, 24 cm; section thickness, 1.2 mm; and resolution, 1.0 × 1.0 × 1.2 mm. All images were corrected for distortion due to gradient non-linearity using Grad Warp software [17] and for intensity inhomogeneity using the “N3” function [18].

#### Image processing for brain volume*.*

The analysis was conducted using the Computational Anatomy Toolbox (CAT12) (C. Gaser, Structural Brain Mapping Group, Jena University Hospital, Jena, Germany; http://dbm.neuro.uni-jena.de/cat/) implemented in Statistical Parametric Mapping 12 (SPM12) software (Wellcome Trust Center for Neuroimaging, London, UK; http://www.fil.ion.ucl.ac.uk/spm/software/spm12/). The 3D-T1-weighted images in the native space were spatially normalized, segmented into GM, white matter (WM), and cerebrospinal fluid, and modulated using the diffeomorphic anatomical registration through the exponential Lie algebra toolbox. To preserve the GMV within each voxel, the images were modulated using the Jacobian determinants derived from spatial normalization. The resulting modulated GM images were smoothed using an 8-mm full-width-at-half-maximum Gaussian kernel. The total intracranial volume (ICV), GMV, and WM volume (WMV) were calculated. The GMV/ICV ratio was reported instead of absolute volume to adjust for individual differences in head size and provide head size-corrected brain volume measures [19,20]. GMV and WMV included both the supratentorial and infratentorial regions. The GMV/ICV and WMV/ICV ratios were also calculated as indicators of global GM and WM atrophy, respectively.

#### Image processing for SBM.

SBM analysis was performed using the Group ICA of fMRI Toolbox (GIFT http://trendscenter.org/software/gift/) [16] in this study. The minimum description length (MDL) principle was used to estimate the number of independent components. MDL identified 147 reliable, independent components (GM structural networks). ICA was performed using a neural network algorithm (Infomax) that attempted to minimize the mutual information of network outputs to identify natural groupings and maximally independent sources [21]. ICAs were repeated 20 times using ICASSO [22] (http://research.ics.aalto.fi/ica/icasso/), and the resulting components were clustered to ensure consistency and reliability. From the GM structural networks generated with ICA, two experienced neuroradiologists (S.K. with 24 years of experience and K.W. with 12 years of experience) independently identified GM structural networks related to DMN. This identification was performed by visually inspecting each component against established patterns documented in prior research, specifically the atlases of brain networks tailored for an elderly cohort (Atlas55+) [23]. Specifically, the anatomical criteria for identifying the DMN included the prominent inclusion of the medial prefrontal cortex and anterior cingulate cortex for the anterior DMN, and the posterior cingulate cortex, precuneus, inferior parietal cortex, and lateral temporal cortices for the posterior DMNs. The inter-rater agreement for the selection of the DMN-related components between the two neuroradiologists was 100%.

The connectivity of the GM structural networks was assessed using loading coefficients, which were standardized as mean Z-scores. Therefore, according to the methods described in a previous study [16], independent components were extracted from 2044 participants. The preprocessed images from these 2044 participants were arrayed into a 2044-row participant-by-GM data matrix. This matrix was comprised of 2044 rows (2044 participants), with each column indicating a voxel. The 2044-row participant-by-GM data matrix was further decomposed into two matrices using ICAs. The first matrix was named the mixing matrix and comprised one participant per row and independent components per column. The mixing matrix involved “loading coefficients,” demonstrating how each structural component contributed to the 2044 participants and thus contained information about the relationship between each participant and each component. The second matrix was called the source matrix, and the relationship between the ICs and voxels was specified. For GMV component visualization, the source matrix was reshaped back into a 3D image and converted into a Z-score map by scaling voxel values to unit standard deviation across the whole brain. These Z maps represent the relative contribution of each voxel to the component. To highlight regions with substantial loadings, the maps were thresholded at Z > 2.0, which approximately corresponds to p < 0.05 (two-tailed) under the standard normal distribution assumption.

### Statistical analyses

All statistical analyses were performed using the EZR (Easy R) software (Saitama Medical Center, Jichi Medical University, Saitama, Japan) [24]. Nominal variables, including the presence or absence of diabetes, hypertension, and hyperlipidemia, were expressed as counts and percentages. Continuous variables were summarized as the mean ± standard deviation, median [interquartile range], or range based on their distribution.

Spearman’s correlation analyses were conducted separately to evaluate the unadjusted associations between plasma vitamin C levels and GMV/ICV, WMV/ICV, and each of the three DMN components. The full correlation matrix, including all clinical and demographic variables, is provided in S1 Table. Multiple regression analysis was used to investigate the independent associations between brain volume measurements (GMV/ICV ratio as an indicator of global GM atrophy and WMV/ICV ratio as an indicator of global WM atrophy), DMN connectivity, and plasma vitamin C levels after adjusting for potential confounders. Assumptions for the multiple regression models were formally assessed prior to interpretation. Multicollinearity among independent variables was evaluated using the Variance Inflation Factor (VIF); all VIF values were below 3, indicating no significant multicollinearity. Additionally, the normality and homoscedasticity of the residuals were confirmed through visual inspection of diagnostic plots.

We clarified that continuous variables (age, Mini-Mental State Examination score [MMSE], brain volume ratios, and plasma vitamin C levels) were z-scored before regression analysis to obtain standardized beta coefficients. Categorical variables (sex, education levels, diabetes, hypertension, and hyperlipidemia, smoking history, drinking history, and physical activit**y**) were treated as dummy variables and were not z-scored, as this would be statistically inappropriate for categorical data. These analyses were adjusted for age, sex, education level (less than high school, high school or equivalent, college or graduate, or professional school), MMSE, self-reported medical history (diabetes, hypertension, and hyperlipidemia), and lifestyle factors (smoking history, drinking history, and physical activity). To account for multiple comparisons across the three DMN components, False Discovery Rate (FDR) correction was applied using the Benjamini-Hochberg method, and a q-value of <0.05 was considered statistically significant. Standardized β-coefficients were reported to represent the relative strength of associations between plasma vitamin C levels and each outcome variable on a comparable scale. A higher absolute value of the standardized β-coefficient indicates a stronger association. Standard errors (SEs) of the standardized β-coefficients were also calculated to indicate the precision and reliability of the estimated effects. A p-value of <0.05 was considered statistically significant.

### Voxel-wise VBM analysis

To provide additional anatomical specificity, statistical analyses were performed using SPM12 software. Morphological changes in regional gray matter volume associated with plasma vitamin C levels were assessed using a multiple regression model, as described in a previous study [25]. The identical clinical and demographic variables used in the SBM analysis, along with lifestyle factors (smoking history, drinking history, and physical activity) and total intracranial volume, were included as covariates of no interest into all analyses to control for confounding variables. Statistical significance was set at voxel-level family-wise error (FWE)-corrected p < 0.05 across the whole brain, with no additional extent threshold for statistical inference.

## Results

**Fig 1:**
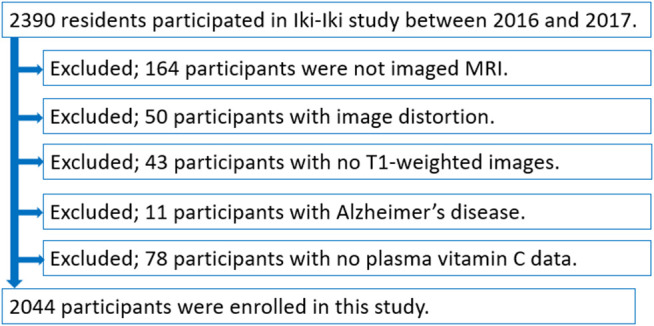
Participant flow chart. A total of 2,390 community-dwelling residents participated in the Iki-Iki study. Of these, 346 participants were excluded due to missing data or other reasons. Consequently, 2,044 participants were included in the analysis.

Of the 2,390 participants of the Iki-Iki Health Promotion Project, 2,226 (93.1%) participants underwent brain MRI. We excluded 50 participants with MRI showing image distortions (n = 50: 7 participants with metal artifacts, 13 participants with excessive motion artifacts, and 30 participants for whom brain volume or structural covariance intra-networks could not be measured accurately for various reasons). Forty-three participants without T1-weighted images were also excluded. Furthermore, we excluded participants with AD (n = 11), because the aim of this study was to emphasize the importance of vitamin C intake in older adults. Among the remaining 2,122 participants, we further excluded participants with missing data on plasma vitamin C levels or lifestyle factors (smoking, alcohol consumption, and physical activity). Consequently, 2,044 participants were included in this study (Fig 1).

The general characteristics of the study participants are summarized in Table 1. The mean age was 70.0 ± 4.2 years (median 69, interquartile range [IQR]: 67−73, 95% CI: 69.8–70.2), with a female-to-male ratio of 1.57:1 (1,248 females [61.1%], 796 males [38.9%]). There were 260 participants (12.7%) with diabetes, 948 (46.4%) with hypertension and 899 (44.0%) with hyperlipidemia. Furthermore, regarding lifestyle factors, 685 participants (33.5%) had a smoking history, 1076 participants (52.6%) had a drinking history, and 1,422 participants (69.6%) engaged in regular physical activity. The GMV/ICV ratio and the WMV/ICV ratio were 0.42 [0.40–0.44] and 0.33 [0.31–0.34], respectively (Table 1).

**Table 1:** Participant characteristics (n = 2,044).

Characteristic	Value
Age (years)	69 [67-73]
Sex: male/female	796/1,248
Education: Junior high school/High school or higher	364/1,680
MMSE score	28[26–30]
Diabetes	260 (12.7%)
Hypertension	948 (46.4%)
Hyperlipidemia	899 (44.0%)
Smoking history	685 (33.5%)
Drinking history	1,076 (52.6%)
Physical activity	1,422 (69.6%)
GMV/ICV ratio	0.42 [0.40-0.44]
WMV/ICV ratio	0.33 [0.31-0.34]
Plasma vitamin C levels, μg/ml	7.38 [5.59-11.57]

Data are presented as n (%) or the median [IQR].

Abbreviations: MMSE, Mini-Mental State Examination; ICV, intracranial volume; GMV, gray matter volume; WMV, white matter volume.

### Global brain volumes

**Fig 2:**
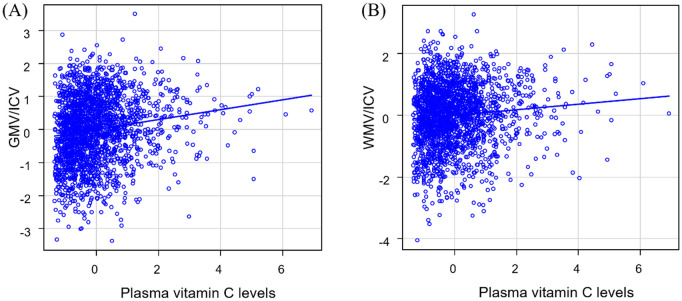
Scatter plots showing the association between plasma vitamin C levels and brain volumes. Standardized values were used to examine the associations between plasma vitamin C levels and the following dependent variables: **(A)** GMV/ICV, **(B)** WMV/ICV. Spearman’s rank correlation coefficients (ρ) and *p*-values were as follows: (A) ρ = 0.196, *p* < 0.001; (B) ρ = 0.103, *p* < 0.001.

After adjusting for potential confounders, the presence of diabetes (β = −0.240 [SE = 0.054], p < 0.001) was significantly associated with the GMV/ICV ratio, while a smoking history (β = −0.121 [SE = 0.055], p = 0.027) was significantly associated with the WMV/ICV ratio (Table 2). Plasma vitamin C levels were significantly associated with both the GMV/ICV ratio (β = 0.076 [SE = 0.018], p < 0.001) and the WMV/ICV ratio (β = 0.074 [SE = 0.020], p < 0.001). Spearman’s correlation analyses revealed a positive correlation between plasma vitamin C levels and GMV/ICV (ρ [Spearman’s rank correlation coefficient] = 0.196, p < 0.001), as well as with WMV/ICV (ρ = 0.103, p < 0.001) (Fig 2).

**Table 2:** Associations between brain volume and plasma vitamin C levels.

	GMV/ICV ratio		WMV/ICV ratio	
	β (SEs)	p	β (SEs)	p
Age	−0.332 (0.019)	<0.001	−0.463 (0.021)	<0.001
Sex	0.857 (0.051)	<0.001	0.060 (0.057)	0.295
Education: Junior high school	0.241 (0.055)	<0.001	0.080 (0.062)	0.197
Education: High school or higher	0.133 (0.040)	0.001	0.053 (0.045)	0.242
MMSE	0.017 (0.008)	0.034	0.001 (0.009)	0.868
Diabetes	−0.240 (0.054)	<0.001	−0.084 (0.060)	0.160
Hypertension	−0.016 (0.036)	0.664	−0.025 (0.040)	0.598
Hyperlipidemia	0.070 (0.036)	0.052	0.021 (0.040)	0.598
Smoking history	−0.035 (0.049)	0.477	−0.121 (0.055)	0.027
Drinking history	−0.082 (0.042)	0.048	−0.018 (0.047)	0.706
Physical activity	0.049 (0.039)	0.209	−0.003 (0.043)	0.938
Plasma vitamin C levels	0.076 (0.018)	<0.001	0.074 (0.020)	<0.001

β (SEs) = β-coefficient (standard errors).

### Source-based morphometry (SBM) analysis

**Fig 3:**
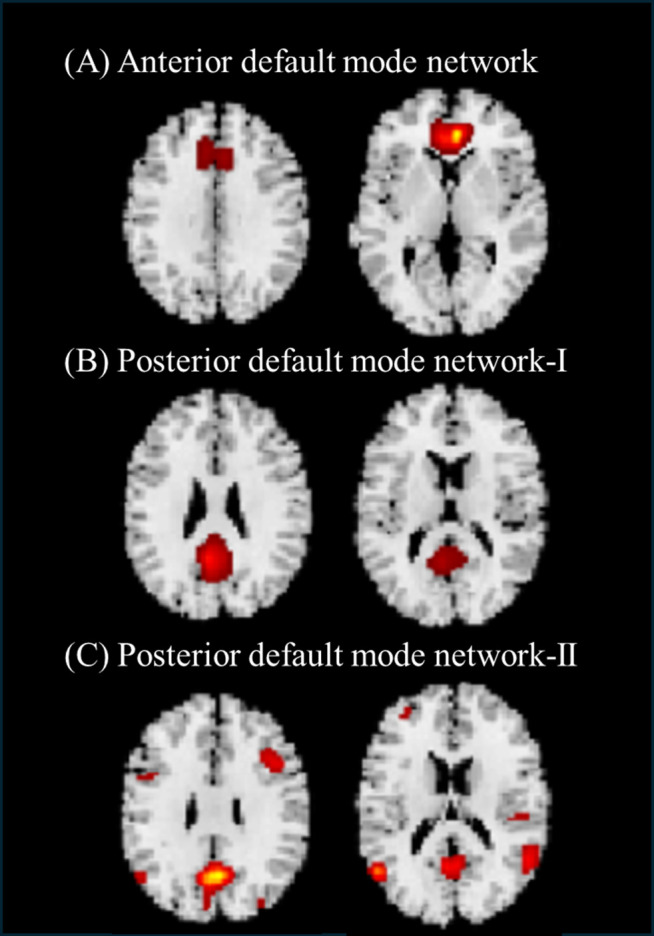
Three GM structural networks related to DMN. Three GM structural networks related to DMN that were identified are shown. (A) anterior DMN; (B) posterior DMN-I, including the PCC and precuneus; and (C) posterior DMN-II, including the PCC, precuneus, inferior parietal cortex, and lateral temporal cortices.

**Fig 4:**
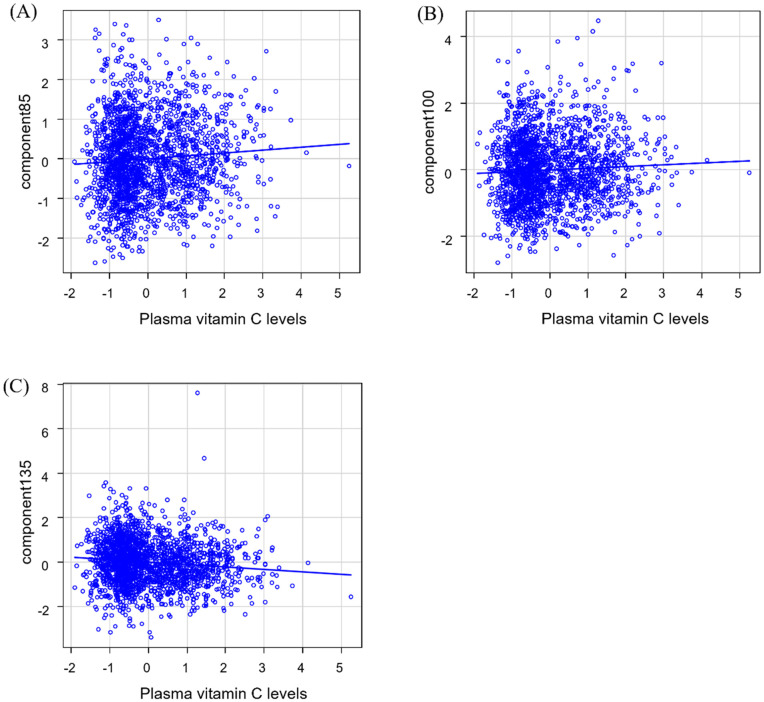
Scatter plots showing the association between plasma vitamin C levels and DMN connectivity. Standardized values were used to examine the associations between plasma vitamin C levels and the following dependent variables: (A) anterior DMN (component 85), (B) posterior DMN-I (component 100), and (C) posterior DMN-II (component 135). Spearman’s rank correlation coefficients (ρ) and *p*-values were as follows: (A) ρ = 0.064, *p* < 0.01; (B) ρ = 0.034, *p* = 0.123; (C) ρ = −0.122, *p* < 0.001.

The ICA generated 147 GM structural networks. Of the 147 GM structural networks, we identified three networks (Components 85, 100, and 135) as the anterior DMN; posterior DMN-I, including the PCC and precuneus; and posterior DMN-II, including the PCC, precuneus, inferior parietal cortex, and lateral temporal cortices, respectively (Fig 3). Plasma vitamin C levels were significantly associated with all three DMN components, although the presence of diabetes and hypertension were significantly associated with posterior DMN-I, and a smoking history was significantly associated with the anterior DMN (Table 3). The standard partial regression coefficients (β) for plasma vitamin C levels in relation to the dependent variables of the anterior DMN, posterior DMN-I, and posterior DMN-II were β = 0.097 (SE = 0.022), p < 0.001; β = 0.082 (SE = 0.022), p < 0.001; and β = −0.111 (SE = 0.022), p < 0.001, respectively, indicating significant independent associations. After FDR correction for multiple comparisons, the associations between plasma vitamin C levels and all three DMN components remained highly statistically significant (all FDR-adjusted q < 0.001). Regarding the DMN components, a significant positive correlation was observed for component 85 (ρ = 0.064, p < 0.01), while component 100 showed no significant association (ρ = 0.034, p = 0.123). Notably, component 135 exhibited a negative correlation with plasma vitamin C levels (ρ = −0.122, p < 0.001) (Fig 4).Furthermore, unadjusted Spearman correlation analyses revealed that MMSE scores were not directly correlated with plasma vitamin C levels (ρ = −0.011, p = 0.631) (S1Table). However, regarding the DMN components, MMSE scores exhibited significant positive correlations with the anterior DMN (Component 85; ρ = 0.088, p < 0.001) and posterior DMN-I (Component 100; ρ = 0.052, p = 0.018), while no significant correlation was found with the posterior DMN-II (Component 135; ρ = −0.038, p = 0.086).

**Table 3:** Associations between brain voxel-based structural networks and participant characteristics.

	Anterior DMN			Posterior DMN-I			Posterior DMN-II		
	β (SEs)	p	q	β (SEs)	p	q	β (SEs)	p	q
Age	−0.249 (0.022)	< 0.001	–	−0.102 (0.023)	< 0.001	–	0.321 (0.022)	< 0.001	–
Sex	−0.420 (0.062)	< 0.001	–	−0.452 (0.064)	< 0.001	–	0.205 (0.062)	< 0.001	–
Education: Junior high school	0.007 (0.067)	0.918	–	−0.100 (0.068)	0.143	–	0.053 (0.066)	0.491	–
Education: High school or higher	−0.027 (0.049)	0.577	–	−0.069 (0.068)	0.143	–	0.076 (0.048)	0.119	–
MMSE	0.019 (0.009)	0.051	–	0.016 (0.010)	0.090	–	0.005 (0.009)	0.562	–
Diabetes	−0.107 (0.065)	0.100	–	−0.169 (0.045)	0.011	–	0.114 (0.064)	0.077	–
Hypertension	−0.059 (0.044)	0.175	–	−0.137 (0.045)	0.002	–	−0.047 (0.043)	0.277	–
Hyperlipidemia	0.000 (0.043)	0.995	–	−0.007 (0.044)	0.879	–	−0.018 (0.043)	0.684	–
Smoking history	−0.133 (0.059)	0.024	–	−0.096 (0.061)	0.114	–	0.111 (0.059)	0.059	–
Drinking history	−0.079 (0.050)	0.119	–	−0.054 (0.052)	0.299	–	0.040 (0.050)	0.425	–
Physical activity	0.010 (0.047)	0.829	–	0.062 (0.048)	0.197	–	−0.024 (0.046)	0.610	–
Plasma vitamin C levels	0.097 (0.022)	< 0.001	< 0.001	0.082 (0.022)	< 0.001	< 0.001	−0.111 (0.022)	< 0.001	< 0.001

β (SEs) = β-coefficient (standard errors).

q values were calculated using the Benjamini-Hochberg False Discovery Rate (FDR) correction method for multiple comparisons across the three DMN components.

### Voxel-wise VBM analysis

**Fig 5:**
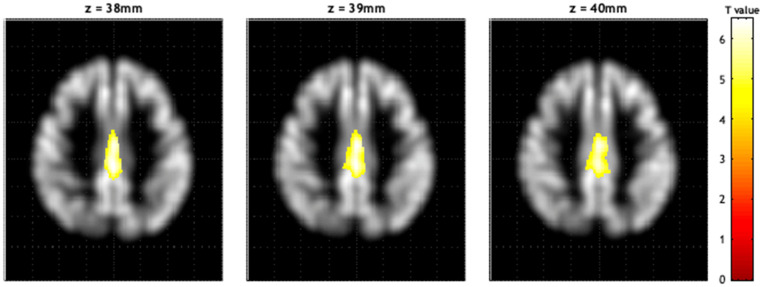
Voxel-wise positive correlation between plasma vitamin C levels and regional gray matter volume. Significant clusters were primarily located in the posterior cingulate cortex, middle cingulate cortex, medial prefrontal cortex, and inferior temporal regions. The statistical parametric map was generated using a multiple regression model adjusted for age, sex, education, Mini-Mental State Examination (MMSE) score, history of hypertension, hyperlipidemia, and diabetes, lifestyle factors (smoking history, drinking history, and physical activity), and total intracranial volume. Statistical significance was set at voxel-level family-wise error (FWE)-corrected p < 0.05 across the whole brain, with no additional extent threshold for statistical inference. The color bar indicates the T-score.

In the voxel-wise VBM analysis, regional gray matter volumes in several brain regions were significantly and positively correlated with plasma vitamin C levels. A significant positive association was observed in the posterior cingulate cortex (peak MNI coordinates: 0, −33, 36; peak T = 6.55; voxel-level P_FWE-corr < 0.001). Other significant clusters were also observed in the middle cingulate cortex, medial prefrontal cortex, and inferior temporal gyri (Table 4 and Fig 5). These localized regions of structural preservation spatially overlapped with the posterior cingulate cortex, a core region of the DMN identified in the SBM analysis.

**Table 4:** Brain regions showing significant positive correlations between regional gray matter volume and plasma vitamin C levels.

Anatomical regions (Neuromorphometrics)	Voxel-level P_FWE -corr	Cluster size (voxels)	Peak-level T-value	MNI coordinates (x, y, z)
Posterior cingulate cortex	< 0.001	1447	6.55	0, −33, 36
Left middle cingulate cortex	0.005	178	4.97	−8, 27, 30
Left inferior temporal gyrus	0.012	89	6.48	−48, −26, −30
Right medial orbital gyrus	0.013	79	5.24	12, 26, −26
Right inferior temporal gyrus	0.021	41	5.56	50, −26, −30

Results of the voxel-based morphometry (VBM) analysis using a multiple regression model. The model was adjusted for age, sex, education, Mini-Mental State Examination (MMSE) score, history of hypertension, hyperlipidemia, and diabetes, lifestyle factors (smoking history, drinking history, and physical activity), and total intracranial volume. Statistical significance was set at voxel-level family-wise error (FWE)-corrected p < 0.05 across the whole brain, with no additional extent threshold for statistical inference. Anatomical regions were identified using the Neuromorphometrics atlas. MNI, Montreal Neurological Institute.

## Discussion

The current study shows a significant association between plasma vitamin C levels and MRI findings after adjusting for potential confounders. Plasma vitamin C levels were independently associated with brain volume. Although our GM structural networks were calculated from brain morphological imaging, previous studies have suggested that altered structures reflect a different pathology from brain atrophy [26]. To the best of our knowledge, this is the first study to demonstrate the association between plasma vitamin C levels and DMN connectivity.

GM and WM volumetric analyses can help detect brain abnormalities and identify the related etiological factors. Specifically, this technique has been sensitive to changes in the GMV due to gross neuronal loss and atrophy [27,28]. Croll et al. reported that a high intake of vegetables, fruits, whole grains, nuts, dairy, and fish and a low intake of sugar-containing beverages were associated with larger brain volume [29]. In another longitudinal study, participants with high vegetable intake had significantly decreased GMV, particularly in the temporal region, over 4 years [30]. Although previous studies have focused on the effects of vegetable intake on brain morphometry, only one study [13] has reported an association between brain volume and plasma vitamin C levels. The study demonstrated that decreased GMV is associated with reduced plasma vitamin C levels in conjunction with increased levels of homocysteine, cholesterol, and LDL [13]. This previous observation may be in line with our results, showing that the plasma vitamin C levels were significantly associated with the GMV/ICV ratio and the WMV/ICV ratio.

Research on DMN connectivity has demonstrated that the DMN plays a significant role in brain health. DMN connectivity has been extensively studied in various diseases. Decreased DMN connectivity is a clinical manifestation of cognitive impairment in patients with AD [31] and mild cognitive impairment [32]. Furthermore, alterations in DMN connectivity have been implicated in depression [33]. DMN connectivity is also altered in schizophrenia, with decreased activation in areas such as the ventromedial prefrontal cortex and the left superior temporal gyrus [34]. Other studies [35–37] have indicated alterations in DMN connectivity, emphasizing its crucial role in schizophrenia pathology. In PD, resting-state fMRI showed decreased DMN connectivity in cognitively unimpaired patients, and this was linked to cognitive outcomes rather than disease progression or treatment without significant GM changes. These findings may highlight DMN connectivity in neurological disorders [38].

Plasma vitamin C levels are also significantly associated with cognitive performance, suggesting that adequate plasma vitamin C levels may support cognitive functions and potentially counteract cognitive decline [39,40]. Furthermore, the association between plasma vitamin C levels and mental health, especially depression, has indicated an inverse correlation where lower blood vitamin C levels were associated with an increased prevalence of depressive symptoms [41,42]. Therefore, plasma vitamin C levels and DMN connectivity may be associated with the pathophysiology of various diseases. These previous findings may support our results, which indicated a link between the plasma vitamin C levels and DMN connectivity. It is important to note that vitamin C may be associated with GM structural networks even in older healthy adults. Interestingly, our results demonstrated an opposing directionality among the DMN components: plasma vitamin C levels were positively associated with the anterior DMN (Component 85) and posterior DMN-I (Component 100), but negatively associated with the posterior DMN-II (Component 135). This apparent discrepancy can be biologically interpreted through their distinct relationships with aging. In our dataset, Components 85 and 100 exhibited negative correlations with age (S1 Table), representing typical structural networks that naturally decline with aging. Conversely, Component 135 showed a strong positive correlation with age, indicating an age-related structural alteration pattern that becomes more pronounced in older adults. Previous neuroimaging studies have established that DMN subsystems undergo heterogeneous and opposing changes during aging, with some networks demonstrating accelerated decline and others exhibiting relative increases or shifting connectivity patterns [43,44]. Therefore, the positive associations with Components 85 and 100 suggest that vitamin C may help preserve normal DMN integrity, whereas the negative association with Component 135 suggests that vitamin C might effectively suppress the progression of age-related aberrant structural network changes.

Furthermore, the voxel-wise VBM analysis successfully provided anatomical specificity, revealing that higher plasma vitamin C levels were significantly associated with preserved regional gray matter volume in regions spatially overlapping with the posterior cingulate cortex, a core node of the DMN. Importantly, these localized structural associations remained robust even after adjusting for comprehensive lifestyle factors (smoking history, drinking history, and physical activity) and total intracranial volume in our large cohort (n = 2044). This voxel-wise anatomical evidence strongly bolsters our SBM findings and reinforces the biological interpretation that optimal vitamin C status may contribute to the structural maintenance of the DMN, independent of major lifestyle-related residual confounders.

To further clarify the clinical implications of our findings, we additionally evaluated the relationships between these variables and cognitive performance. Although plasma vitamin C levels were not directly correlated with MMSE scores, the extracted DMN components (specifically the anterior DMN and posterior DMN-I) were positively correlated with MMSE scores. Furthermore, the adjusted multiple regression models demonstrated that the MMSE score showed a trend toward an independent association with anterior DMN connectivity (p = 0.051). These findings suggest that the structural networks associated with optimal vitamin C status are indeed linked to general cognitive performance in older adults, thereby underscoring the clinical and public health relevance of our neuroimaging results. Humans cannot synthesize vitamin C; thus, it must be obtained exclusively through the dietary intake of fruits and vegetables such as citrus fruits, berries, tomatoes, potatoes, and green leafy vegetables. Our results generate the hypothesis that maintaining sufficient vitamin C status might be beneficial for brain health among older adults, highlighting the need for future longitudinal or interventional studies. This is supported by many previous studies showing the potential protective effect of vitamin C against cognitive decline and depression [40,45,46]. A prospective observational study highlighted that the combined intake of vitamins E (400 IU daily) and C (500 mg daily) for at least 3 years may reduce AD prevalence and incidence [47]. A systematic review and meta-analysis of randomized controlled clinical trials found no significant improvement in mood status in the overall analysis. However, subgroup analysis showed beneficial effects of vitamin C intake in patients who were not prescribed antidepressants (subclinical depression), tentatively suggesting that vitamin C may produce mood-elevating effects in patients with subclinical depression [48]. Our results from this population-based study further highlight the association between vitamin C status and brain networks, generating the hypothesis that promoting effective vitamin C intake may potentially contribute to healthy brain aging in older adults. Although the associations between plasma vitamin C levels, GMV, and DMN connectivity were statistically significant, the effect sizes (standardized β and correlation coefficients of approximately 0.1) were relatively modest. However, the contribution of a single nutritional or lifestyle factor to overall brain structure and large-scale networks is generally small in community-dwelling cohorts. Our effect sizes are not only comparable to those reported in other large cohort studies investigating the relationship between nutrition and brain MRI markers [29,30], but also reach a magnitude similar to that of established major lifestyle risk factors. For instance, a recent large-scale MRI study in a healthy Japanese cohort demonstrated that the effect sizes of established vascular and metabolic risk factors on gray matter volume—such as systolic blood pressure (r = −0.10) and fasting blood glucose (r = −0.12)—are similarly modest [49]. Furthermore, in massive epidemiological datasets such as the UK Biobank (N > 9,000), the effect sizes of individual risk factors including smoking and hypertension on global brain volumes are reported to be on a similar scale (standardized β ranging from approximately 0.05 to 0.10) [50]. From a public health perspective, even such small associations can be clinically meaningful at the population level [51]. The life-long accumulation of adequate nutritional status, such as optimal vitamin C levels, may play a supportive role in preserving cognitive reserve and mitigating age-related brain atrophy and network alterations [52]. Humans cannot synthesize vitamin C; thus, it must be obtained exclusively through the dietary intake of fruits and vegetables such as citrus fruits, berries, tomatoes, potatoes, and green leafy vegetables.

This study has some limitations. First, the cross-sectional design of our study precludes any causal inferences regarding the relationship between plasma vitamin C status and structural DMN connectivity. To establish causality and observe temporal changes, further longitudinal investigations are necessary. Second, only a single plasma vitamin C measurement was obtained, as the blood sample volume allocated for vitamin C analysis was limited to a single draw per participant. Given the known variability of vitamin C levels, this single measurement precludes the estimation of measurement reproducibility or accuracy. However, to minimize short-term dietary effects, all blood samples were collected after an overnight fast. Because humans cannot synthesize vitamin C and rely exclusively on dietary intake, fasting effectively halts the acute supply from meals. Although fasting-induced stress could potentially influence blood levels to some extent, we consider that our measurements reflect the individuals’ steady-state plasma vitamin C concentrations with minimal acute dietary influence. Therefore, measurement errors may have resulted in an underestimation of the association between plasma vitamin C levels and DMN connectivity, and future studies with repeated measurements are warranted. Third, because our cohort consisted exclusively of older Japanese residents with a relatively high educational background, the current findings may not be directly applicable to populations of diverse ethnicities or varying socioeconomic contexts. Finally, while we accounted for major lifestyle factors including smoking, alcohol consumption, and physical activity in our adjusted models, this study did not account for several other important lifestyle and nutritional factors, such as total dietary intake, body mass index (BMI), and socioeconomic status beyond education. These unmeasured variables may act as potential residual confounders. For example, recent neuroimaging studies have demonstrated that higher BMI and obesity are independently associated with reduced connectivity in the posterior DMN [53,54]. Future longitudinal studies incorporating comprehensive lifestyle and anthropometric assessments are needed to clarify the independent contribution of vitamin C to brain structural networks.

## Conclusion

Plasma vitamin C levels are significantly and independently associated with DMN connectivity and GMV after adjusting for potential confounders. The findings highlight the potential importance of plasma vitamin C levels in brain health and suggest the need for further research to explore the underlying mechanisms and potential therapeutic implications.

## Supporting information

S1 Table Unadjusted Spearman correlation matrix among clinical, demographic, and brain MRI variables. (DOCX)
