# Supplementary material for: Plasma vitamin C levels are associated with brain structural networks on MRI: A large cohort study
Source: PLoS One. 2026 Jun 10;21(6):e0348504. doi: 10.1371/journal.pone.0348504 (PMC13252722; doi:10.1371/journal.pone.0348504)
Supplement: S1 Table — (DOCX) [file pone.0348504.s001.docx]

**Supplementary Table S1. Unadjusted Spearman correlation matrix among clinical, demographic, and brain MRI variables**

Data are presented as Spearman's rank correlation coefficients (ρ). *p < 0.05, **p < 0.01, ***p < 0.001. Abbreviations: MMSE, Mini-Mental State Examination; GMV, gray matter volume; WMV, white matter volume; ICV, intracranial volume; Component 85, anterior default mode network (DMN); Component 100, posterior DMN-I; Component 135, posterior DMN-II.

|  | component85 | component100 | component135 | Age | Sex | Education | MMSE | Diabetes | Hypertension | Hyperlipidemia | Smoking history | Drinking history | Physical activity | Vitamin C | GMV/ICV |
| --- | --- | --- | --- | --- | --- | --- | --- | --- | --- | --- | --- | --- | --- | --- | --- |
| component85 |  |  |  |  |  |  |  |  |  |  |  |  |  |  |  |
| component100 | 0.452 *** |  |  |  |  |  |  |  |  |  |  |  |  |  |  |
| component135 | -0.251 *** | -0.103 *** |  |  |  |  |  |  |  |  |  |  |  |  |  |
| Age | -0.244 *** | -0.104 *** | 0.31 *** |  |  |  |  |  |  |  |  |  |  |  |  |
| Sex | -0.0882*** | -0.127 *** | 0.0212 | -0.0414 |  |  |  |  |  |  |  |  |  |  |  |
| Education | 0.067 ** | 0.0779 *** | -0.082 *** | -0.185 *** | -0.0339 |  |  |  |  |  |  |  |  |  |  |
| MMSE | 0.0875 *** | 0.0522 * | -0.0379 | -0.216 *** | 0.0852 *** | 0.22 *** |  |  |  |  |  |  |  |  |  |
| Diabetes | -0.0304 | -0.0404 | 0.0466 * | 0.0445 * | -0.143 *** | -0.0181 | -0.0366 |  |  |  |  |  |  |  |  |
| Hypertension | -0.0494 * | -0.0744 *** | 0.00994 | 0.0865 *** | -0.132 *** | -0.0371 | -0.0227 | 0.144 *** |  |  |  |  |  |  |  |
| Hyperlipidemia | -0.0122 | -0.0216 | -0.0042 | -0.0358 | 0.107 *** | 0.00567 | 0.0339 | 0.101 *** | 0.104 *** |  |  |  |  |  |  |
| Smoking history | 0.0284 | 0.0504 * | 0.0018 | -0.02 | -0.642 *** | 0.0104 | -0.029 | 0.108 *** | 0.115 *** | -0.034 |  |  |  |  |  |
| Drinking history | 0.0432 | 0.0585 ** | -0.0276 | -0.0972 *** | -0.512 *** | 0.0712 ** | -0.00109 | 0.0386 | 0.0844 *** | -0.0498 * | 0.426 *** |  |  |  |  |
| Physical activity | -0.00682 | 0.0229 | 0.00314 | 0.0815 *** | 0.0133 | 0.116 *** | 0.0174 | 0.00995 | 0.0117 | 0.0505 * | -0.0238 | 0.0542 ** |  |  |  |
| Vitamin C | 0.0641 ** | 0.034 | -0.122 *** | -0.0131 | 0.248 *** | 0.015 | -0.0106 | 0.0022 | -0.077 *** | 0.0245 | -0.194 *** | -0.158 *** | 0.0583 ** |  |  |
| GMV/ICV | 0.214 *** | 0.137 *** | -0.0803 *** | -0.332 *** | 0.509 *** | -0.0311 | 0.112 *** | -0.149 *** | -0.104 *** | 0.0922 *** | -0.323 *** | -0.257 *** | -0.00646 | 0.196 *** |  |
| WMV/ICV | 0.323 *** | 0.145 *** | -0.549 *** | -0.459 *** | 0.107 *** | 0.0662 ** | 0.101 *** | -0.0559 * | -0.0665 ** | 0.0418 | -0.0794 *** | -0.0116 | -0.0415 | 0.103 *** | 0.247 *** |
