# Supplementary material for: Plasma vitamin C levels are associated with brain structural networks on MRI: A large cohort study
Source: PLoS One. 2026 Jun 10;21(6):e0348504. doi: 10.1371/journal.pone.0348504 (PMC13252722; doi:10.1371/journal.pone.0348504)
Supplement: Attachment — Submitted filename: Response to Reviewers.docx [file pone.0348504.s003.docx]

**Response to Reviewers**

Dear Dr. Branko Aleksic, Academic Editor, PLOS ONE

We would like to thank you and the reviewers for the thoughtful and constructive comments regarding our manuscript, "Plasma vitamin C levels are associated with brain structural networks on MRI: A large cohort study" (ONE-D-26-01139). We have carefully considered each of the comments and revised the manuscript accordingly. We believe that these revisions have substantially strengthened the interpretative balance, reproducibility, and clinical relevance of our study.

Below, we provide a point-by-point response to the comments from the Academic Editor and the Reviewers. For clarity, the reviewers’ comments are in bold, followed by our responses. All page and line numbers refer to the revised manuscript with track changes.

--------------------------------------------------------------------------------

**Responses to Journal Requirements**

**Journal Requirement #2: Overlapping text** **We noticed you have some minor occurrence of overlapping text with previous publications... In your revision ensure you cite all your sources (including your own works), and quote or rephrase any duplicated text outside the methods section.** **Response:** We sincerely apologize for the overlapping text. In accordance with your instructions, we have carefully reviewed the manuscript and completely rephrased the duplicated sentences outside the Methods section. Specifically, we have thoroughly rewritten the explanation of the source-based morphometry (SBM) technique in the Introduction, as well as the descriptions of the study limitations (regarding the cross-sectional design, sample demographics, and unmeasured confounders) in the Discussion section. We have also ensured that our previous works are appropriately cited. These changes ensure the originality of the text while preserving the intended scientific meaning.

**Journal Requirements #3 and #4: Competing Interests, Funding Statement, and Data Availability** **Response:** We thank the academic editor for the guidance. As requested, we have provided the updated Funding Statement and Competing Interests Statement in our Cover Letter. These statements accurately declare the commercial affiliation, articulate the specific roles of the authors, and explicitly confirm that this commercial affiliation does not alter our adherence to PLOS ONE policies on sharing data and materials. We have also provided a detailed Data Availability Statement specifying the contact information (coi@hirosaki-u.ac.jp) for interested researchers to apply for data access.

**Journal Requirement #5: Ethics Statement** **Response:** In accordance with your instructions, we have moved the ethics statement from the Declarations section to the beginning of the Methods section (now titled "Ethics Statement"), and deleted it from the end of the manuscript.

--------------------------------------------------------------------------------

**Responses to Additional Editor Comments**

**Comment #0-1: Causal language should be tempered given the cross-sectional design, and conclusions regarding maintenance of brain structure or DMN connectivity should be reframed as associative. The magnitude and clinical relevance of the reported effect sizes should be more explicitly discussed.** **Response:** We sincerely appreciate your valuable comments. We have carefully revised the manuscript to temper causal language, reframing our conclusions as associative and hypothesis-generating throughout the Abstract, Discussion, and Conclusion. We have also explicitly discussed the magnitude and clinical relevance of our effect sizes in the revised Discussion. Please see our detailed responses to Reviewer #1-1 and Reviewer #1-2 below for the specific textual changes.

**Comment #0-2: Additional clarification is needed regarding the statistical approach, including the rationale for using both correlation and regression analyses and confirmation that model assumptions were assessed. The identification and interpretation of DMN-related components—particularly the opposing direction observed for one posterior component—require clearer explanation. The limitations section should more explicitly acknowledge potential residual confounding and the use of a single plasma vitamin C measurement. Clarification of the clinical relevance, including the relationship with MMSE, would further strengthen the manuscript.** **Response:** We appreciate your insightful suggestions. We have clarified the rationale for our statistical approach and the verification of regression assumptions (see Response to Reviewer #1-2a). We have provided a clearer biological interpretation of the opposing directionality of Component 135 based on its relationship with aging (see Response to Reviewer #1-3). We have also expanded the limitations section to address potential residual confounding and the single vitamin C measurement (see Responses to Reviewer #1-4 and #1-5). Finally, we have added new analyses demonstrating the relationship between these variables and cognitive performance (MMSE) to strengthen clinical relevance (see Response to Reviewer #2-4).

--------------------------------------------------------------------------------

**Responses to Reviewer #1**

**Comment #1-1: The study is cross-sectional, yet some statements in the Discussion and Conclusion imply a causal role of vitamin C in maintaining brain structure and DMN connectivity. The authors should consistently temper their language and clearly emphasize that causal inferences cannot be made. Statements suggesting “interventions” or “maintaining brain integrity” should be reframed as hypothesis-generating rather than evidence of effect.** **Response:** We sincerely thank the reviewer for this important feedback. We completely agree that causal inferences cannot be drawn from our cross-sectional data. We have carefully revised the manuscript to temper our language throughout the Abstract, Discussion, and Conclusion. Statements that previously implied causality or suggested clinical interventions have been reframed in associative and hypothesis-generating terms. For example, in the Abstract (Conclusions), we modified the text from: *[Original]* "In conclusion, plasma vitamin C levels may be essential for maintaining the structural integrity of the gray matter and for DMN connectivity." *[Revised]* "In conclusion, our findings demonstrate that plasma vitamin C levels are positively associated with the structural integrity of the gray matter and DMN connectivity, generating the hypothesis that vitamin C may play a role in brain health." We have also replaced words such as "predictors" with "independently associated with" throughout the text.

**Comment #1-2: Although several associations reach statistical significance, the standardized β coefficients and correlation coefficients are relatively small. The authors should explicitly discuss the magnitude and potential clinical significance of these effects, rather than focusing primarily on p-values. A brief comparison with effect sizes reported in prior MRI–nutrition studies would strengthen the interpretation.** **Response:** We appreciate this insightful comment. We have added a new paragraph to the Discussion section to explicitly discuss the magnitude and clinical relevance of our findings. Although the effect sizes in our study are relatively modest (standardized β and correlation coefficients of approximately 0.1), they are highly comparable to those reported in other large cohort studies investigating the relationship between nutrition and brain MRI markers. Furthermore, in massive epidemiological datasets (e.g., the UK Biobank), the effect sizes of established major lifestyle risk factors—including smoking and hypertension—on global brain volumes are reported to be similarly modest. Therefore, from a public health perspective, even such small associations can be clinically meaningful at the population level. Additionally, we have updated the p-values to reflect the actual robust significance levels (e.g., p < 0.001) in the revised text, replacing the previous rounded "p < 0.05" reporting.

In the revised manuscript, we have added the following paragraph to the Discussion section:

**[Revised text]** "Although the associations between plasma vitamin C levels, GMV, and DMN connectivity were statistically significant, the effect sizes (standardized β and correlation coefficients of approximately 0.1) were relatively modest. However, the contribution of a single nutritional or lifestyle factor to overall brain structure and large-scale networks is generally small in community-dwelling cohorts. Our effect sizes are not only comparable to those reported in other large cohort studies investigating the relationship between nutrition and brain MRI markers [1] [2], but also reach a magnitude similar to that of established major lifestyle risk factors. For instance, a recent large-scale MRI study in a healthy Japanese cohort demonstrated that the effect sizes of established vascular and metabolic risk factors on gray matter volume—such as systolic blood pressure (r = -0.10) and fasting blood glucose (r = -0.12)—are similarly modest [3]. Furthermore, in massive epidemiological datasets such as the UK Biobank (N > 9,000), the effect sizes of individual risk factors including smoking and hypertension on global brain volumes are reported to be on a similar scale (standardized β ranging from approximately 0.05 to 0.10) [4]. From a public health perspective, even such small associations can be clinically meaningful at the population level [5]. The life-long accumulation of adequate nutritional status, such as optimal vitamin C levels, may play a supportive role in preserving cognitive reserve and mitigating age-related brain atrophy and network alterations [6]. Humans cannot synthesize vitamin C; thus, it must be obtained exclusively through the dietary intake of fruits and vegetables such as citrus fruits, berries, tomatoes, potatoes, and green leafy vegetables."

**Comment #1-2a: The rationale for using both Spearman correlation and multiple regression should be briefly clarified. It would be helpful to specify whether assumptions for regression analyses were formally checked.** **Response:** We have clarified our statistical approach in the revised Methods section. In accordance with standard epidemiological modeling practices, we first used Spearman’s correlation to evaluate the unadjusted, monotonic relationships (the full correlation matrix is now provided in Supplementary Table S1). Subsequently, we employed multiple regression analysis to determine independent associations after adjusting for potential confounders. We also formally assessed the assumptions for the multiple regression models. Multicollinearity was evaluated using the Variance Inflation Factor (VIF), with all values < 3. Furthermore, the normality and homoscedasticity of the residuals were confirmed through visual inspection of diagnostic plots. These methodological details have been added to the *Statistical analyses* section.

In the revised manuscript, we have modified the text as follows:

**[Revised text] (Methods - Statistical analyses)** "Spearman’s correlation analyses were conducted separately to evaluate the unadjusted associations between plasma vitamin C levels and GMV/ICV, WMV/ICV, and each of the three DMN components. The full correlation matrix, including all clinical and demographic variables, is provided in Supplementary Table S1. Multiple regression analysis was used to investigate the independent associations between brain volume measurements (GMV/ICV ratio as an indicator of global GM atrophy and WMV/ICV ratio as an indicator of global WM atrophy), DMN connectivity, and plasma vitamin C levels after adjusting for potential confounders. Assumptions for the multiple regression models were formally assessed prior to interpretation. Multicollinearity among independent variables was evaluated using the Variance Inflation Factor (VIF); all VIF values were below 3, indicating no significant multicollinearity. Additionally, the normality and homoscedasticity of the residuals were confirmed through visual inspection of diagnostic plots."

**Comment #1-3: One DMN component (posterior DMN-II) shows a negative association with plasma vitamin C levels, while others show positive associations. This opposing directionality requires clearer biological interpretation.** **Response:** We agree that a clearer biological interpretation is necessary. We further investigated the relationships between these DMN components and age (Supplementary Table S1). We found that Components 85 and 100 exhibited negative correlations with age, representing typical structural networks that naturally decline with aging. Conversely, Component 135 showed a strong positive correlation with age, indicating an age-related structural alteration pattern that becomes more pronounced in older adults. Therefore, the negative association between plasma vitamin C and Component 135 suggests that optimal vitamin C status might effectively suppress the progression of this age-related aberrant network alteration. We have explicitly added this biological interpretation to the Discussion section.

In the revised manuscript, we have added the following paragraph to the Discussion section:

**[Revised text]** "Interestingly, our results demonstrated an opposing directionality among the DMN components: plasma vitamin C levels were positively associated with the anterior DMN (Component 85) and posterior DMN-I (Component 100), but negatively associated with the posterior DMN-II (Component 135). This apparent discrepancy can be biologically interpreted through their distinct relationships with aging. In our dataset, Components 85 and 100 exhibited negative correlations with age (Supplementary Table S1), representing typical structural networks that naturally decline with aging. Conversely, Component 135 showed a strong positive correlation with age, indicating an age-related structural alteration pattern that becomes more pronounced in older adults. Previous neuroimaging studies have established that DMN subsystems undergo heterogeneous and opposing changes during aging, with some networks demonstrating accelerated decline and others exhibiting relative increases or shifting connectivity patterns [7] [8]. Therefore, the positive associations with Components 85 and 100 suggest that vitamin C may help preserve normal DMN integrity, whereas the negative association with Component 135 suggests that vitamin C might effectively suppress the progression of age-related aberrant structural network changes."

**Comment #1-3a: DMN components were identified by expert neuroradiologists. While acceptable, reporting inter-rater agreement or referencing a standardized atlas-based confirmation would improve reproducibility.**

**Response:** We completely agree that providing these details improves reproducibility. In the revised Methods section (*Image processing for SBM*), we have clarified that two experienced board-certified neuroradiologists independently evaluated the independent components. The inter-rater agreement between the two raters was 100%. Furthermore, this visual identification was guided by cross-referencing the spatial patterns with a standardized brain functional atlas tailored for late adulthood cohorts (Atlas55+) [9].

In the revised manuscript, we have modified the text as follows:

**[Revised text] (Methods - Image processing for SBM)** "From the extracted independent components, two experienced neuroradiologists (S.K. with 24 years of experience, and K.W. with 12 years of experience) independently identified the DMN-related GM structural networks. This identification was performed by visually inspecting each component against established patterns documented in prior research, specifically the atlases of brain networks tailored for an elderly cohort (Atlas55+) [9]. Specifically, the anatomical criteria for identifying the DMN included the prominent inclusion of the medial prefrontal cortex and anterior cingulate cortex for the anterior DMN, and the posterior cingulate cortex, precuneus, inferior parietal cortex, and lateral temporal cortices for the posterior DMNs. The inter-rater agreement for the selection of the DMN-related components between the two neuroradiologists was 100%."

**Comment #1-4: Important lifestyle and nutritional factors (e.g., total dietary intake, physical activity, BMI, smoking, alcohol consumption, and socioeconomic status beyond education) were not included. The authors should explicitly acknowledge them as potential residual confounders.**

**Response:** We sincerely thank the reviewer for this insightful comment. We completely agree that these lifestyle and nutritional factors are crucial. As the reviewer correctly pointed out, smoking, alcohol consumption, and physical activity are recognized as important lifestyle factors influencing both vitamin C metabolism and brain structural networks. For instance, smokers have been consistently reported to exhibit lower serum vitamin C concentrations, attributable to both reduced dietary intake and increased utilization for the neutralization of smoking-induced free radicals [10]. Regarding alcohol consumption, acute intake has been shown to increase urinary excretion of ascorbic acid, whereas prior intake may facilitate alcohol metabolism [11]. Moreover, physical activity may indirectly influence plasma levels through its association with healthier dietary behaviors, including increased consumption of fruits and vegetables [12]. Concurrently, recent neuroimaging studies have demonstrated that physical activity [13], smoking [14] [15], and alcohol consumption [16]significantly alter functional and structural connectivity within large-scale brain networks, including the DMN.

To address this rigorously, we have conducted an updated re-analysis of our data (n=2044) by newly incorporating these three major lifestyle factors (smoking history, drinking history, and physical activity) as covariates in our multiple regression models. Importantly, plasma vitamin C levels remained significantly and independently associated with the DMN components and GMV (all p < 0.001) even after adjusting for these lifestyle factors. We have updated the Methods, Results, Table 1, Table 2, and Table 3 accordingly.

Finally, we explicitly acknowledged the remaining unmeasured factors (e.g., total dietary intake and BMI) as potential residual confounders in the Limitations section. For example, recent neuroimaging studies have demonstrated that higher BMI and obesity are independently associated with reduced connectivity in the posterior DMN [17] [18]. Future longitudinal studies incorporating comprehensive lifestyle and anthropometric assessments are needed to clarify the independent contribution of vitamin C to brain structural networks.

In the revised manuscript, we have modified the text as follows:

**[Revised text] (Methods - Statistical analyses)** "These analyses were adjusted for age, sex, education level (less than high school, high school or equivalent, college or graduate, or professional school), MMSE, self-reported medical history (diabetes, hypertension, and hyperlipidemia), and lifestyle factors (smoking history, drinking history, and physical activity)."

**[Revised text] (Results - Participant characteristics and exclusions)** "... Furthermore, we excluded participants with AD (n=11), because the aim of this study was to emphasize the importance of vitamin C intake in older adults. Among the remaining 2122 participants, we further excluded participants with missing data on plasma vitamin C levels or lifestyle factors (smoking, alcohol consumption, and physical activity). Consequently, 2044 participants were included in this study (Figure 1)." "... with a female-to-male ratio of 1.57:1 (1,248 females [61.1%], 796 males [38.9%]). There were 260 participants (12.7%) with diabetes, 948 (46.4%) with hypertension and 899 (44.0%) with hyperlipidemia. Furthermore, regarding lifestyle factors, 685 participants (33.5%) had a smoking history, 1076 participants (52.6%) had a drinking history, and 1,422 participants (69.6%) engaged in regular physical activity. The GMV/ICV ratio and the WMV/ICV ratio were 0.42 [0.40-0.44] and 0.33 [0.31-0.34], respectively (Table 1)."

**[Revised text] (Discussion - Limitations)** "Finally, while we accounted for major lifestyle factors including smoking, alcohol consumption, and physical activity in our adjusted models, this study did not account for several other important lifestyle and nutritional factors, such as total dietary intake, body mass index (BMI), and socioeconomic status beyond education. These unmeasured variables may act as potential residual confounders. For example, recent neuroimaging studies have demonstrated that higher BMI and obesity are independently associated with reduced connectivity in the posterior DMN [17] [18]. Future longitudinal studies incorporating comprehensive lifestyle and anthropometric assessments are needed to clarify the independent contribution of vitamin C to brain structural networks."

**Comment #1-5: Only a single plasma vitamin C measurement was used. Given the known variability of vitamin C levels, the authors should more clearly discuss how measurement variability and short-term dietary effects might influence results.** **Response:** We agree that plasma vitamin C levels can be variable. However, in our study, all blood samples were collected after an overnight fast. Because humans cannot synthesize vitamin C and rely exclusively on external intake, fasting effectively halts the acute supply from meals. This protocol minimized the acute effects of short-term dietary intake, allowing us to evaluate steady-state (basal) concentrations. Furthermore, the use of a single measurement was unavoidable because the blood sample volume allocated for vitamin C analysis was limited to a single draw per participant in this cohort. We have explicitly discussed these points in the Limitations section of the revised manuscript.

In the revised manuscript, we have modified the text as follows:

**[Revised text] (Discussion - Limitations)** "Second, only a single plasma vitamin C measurement was obtained, as the blood sample volume allocated for vitamin C analysis was limited to a single draw per participant. Given the known variability of vitamin C levels, this single measurement precludes the estimation of measurement reproducibility or accuracy. However, to minimize short-term dietary effects, all blood samples were collected after an overnight fast. Because humans cannot synthesize vitamin C and rely exclusively on dietary intake, fasting effectively halts the acute supply from meals. Although fasting-induced stress could potentially influence blood levels to some extent, we consider that our measurements reflect the individuals' steady-state plasma vitamin C concentrations with minimal acute dietary influence. Therefore, measurement errors may have resulted in an underestimation of the association between plasma vitamin C levels and DMN connectivity, and future studies with repeated measurements are warranted."

--------------------------------------------------------------------------------

**Responses to Reviewer #2**

**Comment #2-1: (The reviewer summarized the strengths of the study and suggested some clarifications to enhance reproducibility and clinical interpretation, particularly regarding the identification of DMN components and the causal language).** **Response:** We sincerely thank the reviewer for the positive evaluation of our study design, sample size, and imaging pipeline. As detailed in our responses to Reviewer #1-1 and #1-3a, we have carefully toned down the causal language throughout the manuscript and have explicitly outlined the anatomical criteria, the use of a standardized atlas (Atlas55+), and the 100% inter-rater agreement between the two neuroradiologists for identifying the DMN components.

**Comment #2-2: Voxel-wise analyses could provide anatomical specificity and strengthen the biological interpretation. The inclusion of a voxel-wise VBM analysis (GM, and possibly WM) to identify regions associated with plasma vitamin C seems feasible.**

**Response:** We sincerely thank the reviewer for this highly constructive suggestion. In accordance with your recommendation, we performed a voxel-wise Voxel-Based Morphometry (VBM) analysis using a multiple regression model, adjusting for age, sex, education, MMSE, hypertension, hyperlipidemia, diabetes, comprehensive lifestyle factors (smoking history, drinking history, and physical activity), and total intracranial volume. The VBM results demonstrated that higher plasma vitamin C levels were significantly associated with preserved regional gray matter volume, primarily located in the posterior cingulate cortex (peak MNI coordinates: 0, -33, 36; voxel-level P_FWE-corr < 0.001), middle cingulate cortex, medial prefrontal cortex, and inferior temporal gyri. These localized regions of structural preservation spatially overlapped with the posterior cingulate cortex, a core region of the DMN identified in our SBM analysis. This voxel-wise anatomical evidence robustly supports our SBM findings. We have added the detailed VBM methods and results to the revised manuscript, included the VBM statistical map as a new Figure 5, and summarized the significant clusters in a new Table 4.

In the revised manuscript, we have added the details of the VBM analysis as follows:

**[Revised text] (Methods - Voxel-wise VBM analysis)** "To provide additional anatomical specificity, statistical analyses were performed using SPM12 software. Morphological changes in regional gray matter volume associated with plasma vitamin C levels were assessed using a multiple regression model, as described in a previous study [19]. The identical clinical and demographic variables used in the SBM analysis, along with lifestyle factors (smoking history, drinking history, and physical activity) and total intracranial volume, were included as covariates of no interest into all analyses to control for confounding variables. Statistical significance was set at voxel-level family-wise error (FWE)-corrected p < 0.05 across the whole brain, with no additional extent threshold for statistical inference."

**[Revised text] (Results - Voxel-wise VBM analysis)** "In the voxel-wise VBM analysis, regional gray matter volumes in several brain regions were significantly and positively correlated with plasma vitamin C levels. A significant positive association was observed in the posterior cingulate cortex (peak MNI coordinates: 0, -33, 36; peak T = 6.55; voxel-level P_FWE-corr < 0.001). Other significant clusters were also observed in the middle cingulate cortex, medial prefrontal cortex, and inferior temporal gyri (Table 4 and Figure 5). These localized regions of structural preservation spatially overlapped with the posterior cingulate cortex, a core region of the DMN identified in the SBM analysis."

**[Revised text] (Discussion)** "Furthermore, the voxel-wise VBM analysis successfully provided anatomical specificity, revealing that higher plasma vitamin C levels were significantly associated with preserved regional gray matter volume in regions spatially overlapping with the posterior cingulate cortex, a core node of the DMN. Importantly, these localized structural associations remained robust even after adjusting for comprehensive lifestyle factors (smoking history, drinking history, and physical activity) and total intracranial volume in our large cohort (n = 2044). This voxel-wise anatomical evidence strongly bolsters our SBM findings and reinforces the biological interpretation that optimal vitamin C status may contribute to the structural maintenance of the DMN, independent of major lifestyle-related residual confounders."

**Comment #2-3: Please reconcile the interpretation of DMN findings (including Component 135). In the Abstract and Discussion, the narrative suggests that plasma vitamin C may be important for “maintaining DMN connectivity”... However, Component 135 shows a negative correlation.**

**Response:** We appreciate this insightful comment. First, we have carefully revised the manuscript to tone down causal implications such as "maintaining," reframing them in associative terms. Second, we have provided a clearer biological interpretation for the negative association with Component 135 in the Discussion section.

In the revised manuscript, we have modified the text as follows:

**[Revised text] (Abstract - Background)** "Neurodegenerative diseases significantly impact brain health in older adults, and although dietary vitamin C intake has been associated with a reduced risk of cognitive impairment, it remains unclear whether plasma vitamin C levels independently affect brain structure and neural connectivity. This study aimed to investigate whether plasma vitamin C levels were **independently associated with** brain volume and default mode network (DMN) connectivity in older adults."

**[Revised text] (Results - Table 3)** *(Regarding Component 135):* Plasma vitamin C levels demonstrated a significant independent negative association with Posterior DMN-II (β = -0.111, SE = 0.022, p < 0.001, q < 0.001).

**Comment #2-4: Given that MMSE data is available, reporting the clinical relevance of the imaging findings would be beneficial. Showing whether the association between vitamin C and DMN-related structural networks is linked to cognition would clarify the clinical implications.**

**Response:** We completely agree that demonstrating the relationship with cognitive performance enhances the clinical relevance of our findings. In the revised manuscript, we have reported the independent associations between MMSE scores and the extracted DMN-related components using a multiple regression model. As detailed in the revised Table 3, the MMSE score showed a trend toward an independent positive association with anterior DMN connectivity (p = 0.051). Furthermore, we have expanded upon these clinical implications in the Discussion section, emphasizing that structural networks associated with optimal vitamin C status are linked to general cognitive performance.

In the revised manuscript, we have added the following text:

**[Revised text] (Results - Table 3. Associations between brain voxel-based structural networks and participant characteristics)**

Anterior DMN: MMSE β = 0.019 (SE = 0.009), p = 0.051

Posterior DMN-I: MMSE β = 0.016 (SE = 0.010), p = 0.090

Posterior DMN-II: MMSE β = 0.005 (SE = 0.009), p = 0.562

(Note: These associations were analyzed using a multiple regression model adjusted for age, sex, education, diabetes, hypertension, hyperlipidemia, smoking history, drinking history, physical activity, and plasma vitamin C levels.)

**[Revised text] (Discussion)** "To further clarify the clinical implications of our findings, we additionally evaluated the relationships between these variables and cognitive performance. Although plasma vitamin C levels were not directly correlated with MMSE scores, the extracted DMN components (specifically the anterior DMN and posterior DMN-I) were positively correlated with MMSE scores. Furthermore, the adjusted multiple regression models demonstrated that the MMSE score showed a trend toward an independent association with anterior DMN connectivity (p = 0.051). These findings suggest that the structural networks associated with optimal vitamin C status are indeed linked to general cognitive performance in older adults, thereby underscoring the clinical and public health relevance of our neuroimaging results."

--------------------------------------------------------------------------------

We hope that these revisions adequately address all of your concerns and that the manuscript is now suitable for publication in PLOS ONE.

Sincerely,

Tomohiro Shintaku, MD, PhD Department of Radiology, Graduate School of Medicine, Hirosaki University 5 Zaifu-cho, Hirosaki, Aomori 036-8562, Japan Email: shintaku@hirosaki-u.ac.jp

**References**

1.	Croll PH, Voortman T, Ikram MA, Franco OH, Schoufour JD, Bos D, et al. Better diet quality relates to larger brain tissue volumes: The Rotterdam Study. Neurology. 2018;90(24):e2166-e73. Epub 20180516. doi: 10.1212/wnl.0000000000005691. PubMed PMID: 29769374.

2.	Lee S, Kim EY, Shin C. Changes in Brain Volume Associated With Vegetable Intake in a General Population. J Am Coll Nutr. 2019;38(6):506-12. Epub 20190321. doi: 10.1080/07315724.2018.1563002. PubMed PMID: 30897041.

3.	Watanabe K, Kakeda S, Nemoto K, Onoda K, Yamaguchi S, Kobayashi S, et al. Effects of Obesity, Blood Pressure, and Blood Metabolic Biomarkers on Grey Matter Brain Healthcare Quotient: A Large Cohort Study of a Magnetic Resonance Imaging Brain Screening System in Japan. J Clin Med. 2022;11(11). Epub 20220525. doi: 10.3390/jcm11112973. PubMed PMID: 35683364; PubMed Central PMCID: PMCPMC9181611.

4.	Cox SR, Lyall DM, Ritchie SJ, Bastin ME, Harris MA, Buchanan CR, et al. Associations between vascular risk factors and brain MRI indices in UK Biobank. Eur Heart J. 2019;40(28):2290-300. doi: 10.1093/eurheartj/ehz100. PubMed PMID: 30854560; PubMed Central PMCID: PMCPMC6642726.

5.	Gu Y, Brickman AM, Stern Y, Habeck CG, Razlighi QR, Luchsinger JA, et al. Mediterranean diet and brain structure in a multiethnic elderly cohort. Neurology. 2015;85(20):1744-51. Epub 20151021. doi: 10.1212/wnl.0000000000002121. PubMed PMID: 26491085; PubMed Central PMCID: PMCPMC4653103.

6.	Barberger-Gateau P, Raffaitin C, Letenneur L, Berr C, Tzourio C, Dartigues JF, et al. Dietary patterns and risk of dementia: the Three-City cohort study. Neurology. 2007;69(20):1921-30. doi: 10.1212/01.wnl.0000278116.37320.52. PubMed PMID: 17998483.

7.	Jones DT, Machulda MM, Vemuri P, McDade EM, Zeng G, Senjem ML, et al. Age-related changes in the default mode network are more advanced in Alzheimer disease. Neurology. 2011;77(16):1524-31. Epub 20111005. doi: 10.1212/WNL.0b013e318233b33d. PubMed PMID: 21975202; PubMed Central PMCID: PMCPMC3198977.

8.	Staffaroni AM, Brown JA, Casaletto KB, Elahi FM, Deng J, Neuhaus J, et al. The Longitudinal Trajectory of Default Mode Network Connectivity in Healthy Older Adults Varies As a Function of Age and Is Associated with Changes in Episodic Memory and Processing Speed. J Neurosci. 2018;38(11):2809-17. Epub 20180213. doi: 10.1523/jneurosci.3067-17.2018. PubMed PMID: 29440553; PubMed Central PMCID: PMCPMC5852659.

9.	Doucet GE, Labache L, Thompson PM, Joliot M, Frangou S. Atlas55+: Brain Functional Atlas of Resting-State Networks for Late Adulthood. Cereb Cortex. 2021;31(3):1719-31. doi: 10.1093/cercor/bhaa321. PubMed PMID: 33188411; PubMed Central PMCID: PMCPMC7869083.

10.	Schectman G, Byrd JC, Gruchow HW. The influence of smoking on vitamin C status in adults. Am J Public Health. 1989;79(2):158-62. doi: 10.2105/ajph.79.2.158. PubMed PMID: 2913833; PubMed Central PMCID: PMCPMC1349925.

11.	Lim DJ, Sharma Y, Thompson CH. Vitamin C and alcohol: a call to action. BMJ Nutr Prev Health. 2018;1(1):17-22. Epub 20181205. doi: 10.1136/bmjnph-2018-000010. PubMed PMID: 33235950; PubMed Central PMCID: PMCPMC7678474.

12.	Pincemail J, Vanbelle S, Degrune F, Cheramy-Bien JP, Charlier C, Chapelle JP, et al. Lifestyle Behaviours and Plasma Vitamin C and β-Carotene Levels from the ELAN Population (Liège, Belgium). J Nutr Metab. 2011;2011:494370. Epub 20110306. doi: 10.1155/2011/494370. PubMed PMID: 21437178; PubMed Central PMCID: PMCPMC3062067.

13.	Ko YW, Kim SM, Kang KD, Han DH. Changes in Functional Connectivity Between Default Mode Network and Attention Network in Response to Changes in Aerobic Exercise Intensity. Psychiatry Investig. 2023;20(1):27-34. Epub 20230125. doi: 10.30773/pi.2022.0245. PubMed PMID: 36721883; PubMed Central PMCID: PMCPMC9890042.

14.	Murray L, Frederick BB, Janes AC. Data-driven connectivity profiles relate to smoking cessation outcomes. Neuropsychopharmacology. 2024;49(6):1007-13. Epub 20240127. doi: 10.1038/s41386-024-01802-9. PubMed PMID: 38280945; PubMed Central PMCID: PMCPMC11039768.

15.	Im JJ, Kim H, Lee JH, Park HJ, Joh HK, Ahn WY. Longitudinal brain connectivity changes associated with successful smoking cessation. Front Psychol. 2025;16:1734803. Epub 20251217. doi: 10.3389/fpsyg.2025.1734803. PubMed PMID: 41479984; PubMed Central PMCID: PMCPMC12753871.

16.	Francis AN, Salloum IM. High-resolution mapping of alcohol-related brain connectivity in adults using 7T fMRI and multivoxel pattern classification. Psychiatry Res Neuroimaging. 2025;352:112046. Epub 20250810. doi: 10.1016/j.pscychresns.2025.112046. PubMed PMID: 40816048.

17.	Beyer F, Kharabian Masouleh S, Huntenburg JM, Lampe L, Luck T, Riedel-Heller SG, et al. Higher body mass index is associated with reduced posterior default mode connectivity in older adults. Hum Brain Mapp. 2017;38(7):3502-15. Epub 20170411. doi: 10.1002/hbm.23605. PubMed PMID: 28397392; PubMed Central PMCID: PMCPMC6867016.

18.	Donofry SD, Stillman CM, Erickson KI. A review of the relationship between eating behavior, obesity and functional brain network organization. Soc Cogn Affect Neurosci. 2020;15(10):1157-81. doi: 10.1093/scan/nsz085. PubMed PMID: 31680149; PubMed Central PMCID: PMCPMC7657447.

19.	Watanabe K, Kakeda S, Yoshimura R, Abe O, Ide S, Hayashi K, et al. Relationship between the catechol-O-methyl transferase Val108/158Met genotype and brain volume in treatment-naive major depressive disorder: Voxel-based morphometry analysis. Psychiatry Res. 2015;233(3):481-7. Epub 20150731. doi: 10.1016/j.pscychresns.2015.07.024. PubMed PMID: 26253436.
