# Supplementary material for: Plasma vitamin C levels are associated with brain structural networks on MRI: A large cohort study
Source: PLoS One. 2026 Jun 10;21(6):e0348504. doi: 10.1371/journal.pone.0348504 (PMC13252722; doi:10.1371/journal.pone.0348504)
Supplement: Peer review history

# Peer review: Decision Letter 0

**DOI:** 10.1371/journal.pone.0348504.r001

**Authors:** Branko Aleksic

12 Feb 2026

PONE-D-26-01139

Plasma vitamin C levels are associated with brain structural networks on MRI: A large cohort study

PLOS One

Dear Dr. Shintaku,

Thank you for submitting your manuscript to PLOS ONE. After careful consideration, we feel that it has merit but does not fully meet PLOS ONE’s publication criteria as it currently stands. Therefore, we invite you to submit a revised version of the manuscript that addresses the points raised during the review process.

Please submit your revised manuscript by Mar 29 2026 11:59PM. If you will need more time than this to complete your revisions, please reply to this message or contact the journal office at plosone@plos.org. When you're ready to submit your revision, log on to https://www.editorialmanager.com/pone/ and select the 'Submissions Needing Revision' folder to locate your manuscript file.

Please include the following items when submitting your revised manuscript:

- A letter that responds to each point raised by the academic editor and reviewer(s). You should upload this letter as a separate file labeled 'Response to Reviewers'.
- A marked-up copy of your manuscript that highlights changes made to the original version. You should upload this as a separate file labeled 'Revised Manuscript with Track Changes'.
- An unmarked version of your revised paper without tracked changes. You should upload this as a separate file labeled 'Manuscript'.

-->If you would like to make changes to your financial disclosure, please include your updated statement in your cover letter. Guidelines for resubmitting your figure files are available below the reviewer comments at the end of this letter.

If applicable, we recommend that you deposit your laboratory protocols in protocols.io to enhance the reproducibility of your results. Protocols.io assigns your protocol its own identifier (DOI) so that it can be cited independently in the future. For instructions see: https://journals.plos.org/plosone/s/submission-guidelines#loc-laboratory-protocols. Additionally, PLOS ONE offers an option for publishing peer-reviewed Lab Protocol articles, which describe protocols hosted on protocols.io. Read more information on sharing protocols at https://plos.org/protocols?utm_medium=editorial-email&utm_source=authorletters&utm_campaign=protocols.

We look forward to receiving your revised manuscript.

Kind regards,

Branko Aleksic, MD, PhD

Academic Editor

PLOS One

**Journal Requirements:**

When submitting your revision, we need you to address these additional requirements.

1. Please ensure that your manuscript meets PLOS ONE's style requirements, including those for file naming. The PLOS ONE style templates can be found at

https://journals.plos.org/plosone/s/file?id=wjVg/PLOSOne_formatting_sample_main_body.pdf and

https://journals.plos.org/plosone/s/file?id=ba62/PLOSOne_formatting_sample_title_authors_affiliations.pdf

2. We noticed you have some minor occurrence of overlapping text with the following previous publication(s), which needs to be addressed:

- https://doi.org/10.1016/j.acra.2025.04.048

- https://www.ncbi.nlm.nih.gov/pubmed/40531396

In your revision ensure you cite all your sources (including your own works), and quote or rephrase any duplicated text outside the methods section. Further consideration is dependent on these concerns being addressed.

3. Thank you for stating the following in the Competing Interests section:

“I have read the journal's policy and the authors of this manuscript have the following competing interests: A company (KAGOME CO., LTD.) established a department at Hirosaki University. Two authors (Daichi Kokubu and Yusuke Ushida) are employees of the company. Two authors hold stock in the company. This study reflects the views of the scientists, not the company.”

We note that one or more of the authors are employed by a commercial company: KAGOME CO., LTD

1. Please provide an amended Funding Statement declaring this commercial affiliation, as well as a statement regarding the Role of Funders in your study. If the funding organization did not play a role in the study design, data collection and analysis, decision to publish, or preparation of the manuscript and only provided financial support in the form of authors' salaries and/or research materials, please review your statements relating to the author contributions, and ensure you have specifically and accurately indicated the role(s) that these authors had in your study. You can update author roles in the Author Contributions section of the online submission form.

Please also include the following statement within your amended Funding Statement.

“The funder provided support in the form of salaries for authors [insert relevant initials], but did not have any additional role in the study design, data collection and analysis, decision to publish, or preparation of the manuscript. The specific roles of these authors are articulated in the ‘author contributions’ section.”

If your commercial affiliation did play a role in your study, please state and explain this role within your updated Funding Statement.

2. Please also provide an updated Competing Interests Statement declaring this commercial affiliation along with any other relevant declarations relating to employment, consultancy, patents, products in development, or marketed products, etc.

Within your Competing Interests Statement, please confirm that this commercial affiliation does not alter your adherence to all PLOS ONE policies on sharing data and materials by including the following statement: "This does not alter our adherence to  PLOS ONE policies on sharing data and materials.” (as detailed online in our guide for authors http://journals.plos.org/plosone/s/competing-interests) . If this adherence statement is not accurate and  there are restrictions on sharing of data and/or materials, please state these. Please note that we cannot proceed with consideration of your article until this information has been declared.

Please include both an updated Funding Statement and Competing Interests Statement in your cover letter. We will change the online submission form on your behalf.

4. For studies involving third-party data, we encourage authors to share any data specific to their analyses that they can legally distribute. PLOS recognizes, however, that authors may be using third-party data they do not have the rights to share. When third-party data cannot be publicly shared, authors must provide all information necessary for interested researchers to apply to gain access to the data. (https://journals.plos.org/plosone/s/data-availability#loc-acceptable-data-access-restrictions)

For any third-party data that the authors cannot legally distribute, they should include the following information in their Data Availability Statement upon submission:

1) A description of the data set and the third-party source

2) If applicable, verification of permission to use the data set

3) Confirmation of whether the authors received any special privileges in accessing the data that other researchers would not have

4) All necessary contact information others would need to apply to gain access to the data

5. Your ethics statement should only appear in the Methods section of your manuscript. If your ethics statement is written in any section besides the Methods, please move it to the Methods section and delete it from any other section. Please ensure that your ethics statement is included in your manuscript, as the ethics statement entered into the online submission form will not be published alongside your manuscript.

6. If the reviewer comments include a recommendation to cite specific previously published works, please review and evaluate these publications to determine whether they are relevant and should be cited. There is no requirement to cite these works unless the editor has indicated otherwise.

7. Please review your reference list to ensure that it is complete and correct. If you have cited papers that have been retracted, please include the rationale for doing so in the manuscript text, or remove these references and replace them with relevant current references. Any changes to the reference list should be mentioned in the rebuttal letter that accompanies your revised manuscript. If you need to cite a retracted article, indicate the article’s retracted status in the References list and also include a citation and full reference for the retraction notice.

**Additional Editor Comments:**

The study is well designed, leverages a large community-based cohort, and addresses a timely topic. However, several points require clarification to strengthen interpretative balance and reproducibility. Causal language should be tempered given the cross-sectional design, and conclusions regarding maintenance of brain structure or DMN connectivity should be reframed as associative. The magnitude and clinical relevance of the reported effect sizes should be more explicitly discussed.

Additional clarification is needed regarding the statistical approach, including the rationale for using both correlation and regression analyses and confirmation that model assumptions were assessed. The identification and interpretation of DMN-related components—particularly the opposing direction observed for one posterior component—require clearer explanation. The limitations section should more explicitly acknowledge potential residual confounding and the use of a single plasma vitamin C measurement. Clarification of the clinical relevance, including the relationship with MMSE, would further strengthen the manuscript.

These issues are considered minor and primarily involve clarification and refinement of interpretation. We look forward to receiving the revised version.

[Note: HTML markup is below. Please do not edit.]

Reviewers' comments:

Reviewer's Responses to Questions

**Comments to the Author**

1. Is the manuscript technically sound, and do the data support the conclusions?

The manuscript must describe a technically sound piece of scientific research with data that supports the conclusions. Experiments must have been conducted rigorously, with appropriate controls, replication, and sample sizes. The conclusions must be drawn appropriately based on the data presented.

Reviewer #1: Yes

Reviewer #2: Yes

**********

2. Has the statistical analysis been performed appropriately and rigorously?

Reviewer #1: Yes

Reviewer #2: Yes

**********

3. Have the authors made all data underlying the findings in their manuscript fully available?

The PLOS Data policy requires authors to make all data underlying the findings described in their manuscript fully available without restriction, with rare exception (please refer to the Data Availability Statement in the manuscript PDF file). The data should be provided as part of the manuscript or its supporting information, or deposited to a public repository. For example, in addition to summary statistics, the data points behind means, medians and variance measures should be available. If there are restrictions on publicly sharing data—e.g. participant privacy or use of data from a third party—those must be specified.

Reviewer #1: Yes

Reviewer #2: No

**********

4. Is the manuscript presented in an intelligible fashion and written in standard English?

PLOS ONE does not copyedit accepted manuscripts, so the language in submitted articles must be clear, correct, and unambiguous. Any typographical or grammatical errors should be corrected at revision, so please note any specific errors here.

Reviewer #1: Yes

Reviewer #2: Yes

**********

5. Review Comments to the Author

Please use the space provided to explain your answers to the questions above. You may also include additional comments for the author, including concerns about dual publication, research ethics, or publication ethics. (Please upload your review as an attachment if it exceeds 20,000 characters)

Reviewer #1: 1. Causality and interpretation

The study is cross-sectional, yet some statements in the Discussion and Conclusion imply a causal role of vitamin C in maintaining brain structure and DMN connectivity.

The authors should consistently temper their language and clearly emphasize that causal inferences cannot be made.

Statements suggesting “interventions” or “maintaining brain integrity” should be reframed as hypothesis-generating rather than evidence of effect.

2.Effect size and clinical relevance

Although several associations reach statistical significance, the standardized β coefficients and correlation coefficients are relatively small.

The authors should explicitly discuss the magnitude and potential clinical significance of these effects, rather than focusing primarily on p-values.

A brief comparison with effect sizes reported in prior MRI–nutrition studies would strengthen the interpretation.

2a)Statistical clarity

The rationale for using both Spearman correlation and multiple regression should be briefly clarified.

It would be helpful to specify whether assumptions for regression analyses were formally checked.

3.DMN component interpretation

One DMN component (posterior DMN-II) shows a negative association with plasma vitamin C levels, while others show positive associations.

This opposing directionality requires clearer biological interpretation. At present, this finding is described but not adequately explained.

The authors should discuss whether these components represent distinct functional or structural sub-networks and how vitamin C might differentially influence them.

3a)DMN identification

DMN components were identified by expert neuroradiologists. While acceptable, reporting inter-rater agreement or referencing a standardized atlas-based confirmation would improve reproducibility.

4.Potential confounding variables

Important lifestyle and nutritional factors (e.g., total dietary intake, physical activity, BMI, smoking, alcohol consumption, and socioeconomic status beyond education) were not included.

While some of these may not be available, the authors should explicitly acknowledge them as potential residual confounders and discuss how they may influence the observed associations.

5.Plasma vitamin C measurement

Only a single plasma vitamin C measurement was used.

Given the known variability of vitamin C levels, the authors should more clearly discuss how measurement variability and short-term dietary effects might influence results.

Reviewer #2: Overall assessment

This manuscript leverages a large, community-based cohort and combines global volumetric measures (GMV/ICV, WMV/ICV) with ICA-based source-based morphometry (SBM) to examine associations between plasma vitamin C and brain structure in older adults. The sample size and imaging pipeline are clear strengths, and the topic is timely given growing interest in links between nutritional factors and brain health. That said, several methodological and interpretive points would benefit from clarification and, where feasible, additional analyses to improve reproducibility and strengthen the biological and clinical interpretation. In particular, some conclusions regarding “maintenance of DMN connectivity” appear stronger than what is directly supported by the current results, especially considering the negative association observed for one DMN-related component.

Major Comments

1) Clarify the procedure for identifying DMN-related components

Although the authors cite prior studies, the manuscript would benefit from a more detailed description of how DMN-related components were identified among the 147 ICA-derived GM structural networks. The statement that “two neuroradiologists identified DMN-related networks based on previous studies” is helpful, but additional detail is needed to ensure reproducibility. At minimum, please specify the anatomical criteria used to label a component as DMN-related and outline the decision process.

2) Consider adding voxel-wise localization analyses (VBM)

The study reports significant associations between plasma vitamin C levels and global brain volume ratios (GMV/ICV and WMV/ICV). While these global indices are informative, voxel-wise analyses could provide additional anatomical specificity and strengthen the biological interpretation. Since SPM12/CAT12 processing has already been performed, it may be feasible to include voxel-wise VBM analyses (GM and, if appropriate, WM) to localize regions associated with plasma vitamin C.

3) Please reconcile the interpretation of DMN findings (including Component 135)

In the Abstract and Discussion, the narrative suggests that plasma vitamin C may be important for “maintaining DMN connectivity” and could be linked to cognition. However, Component 135 (posterior DMN-II) shows a negative correlation with plasma vitamin C, and this point would benefit from clearer interpretation.

4) Strengthen clinical relevance by reporting associations with MMSE

Given that MMSE is available and included as a covariate, the clinical relevance of the imaging findings could be enhanced by reporting:

• the association between plasma vitamin C levels and MMSE (e.g., correlation and/or adjusted regression); and

• the associations between the extracted DMN-related components (Components 85/100/135) and MMSE.

Showing whether the vitamin C–DMN-related structural network associations relate (or do not relate) to cognitive performance would help clarify the clinical implications.

**********

6. PLOS authors have the option to publish the peer review history of their article (what does this mean?). If published, this will include your full peer review and any attached files.

If you choose “no”, your identity will remain anonymous but your review may still be made public.

**Do you want your identity to be public for this peer review?** For information about this choice, including consent withdrawal, please see our Privacy Policy.

Reviewer #1: No

Reviewer #2: **Yes:** Kazuhide Seo

**********

[NOTE: If reviewer comments were submitted as an attachment file, they will be attached to this email and accessible via the submission site. Please log into your account, locate the manuscript record, and check for the action link "View Attachments". If this link does not appear, there are no attachment files.]

To ensure your figures meet our technical requirements, please review our figure guidelines: https://journals.plos.org/plosone/s/figures

You may also use PLOS’s free figure tool, NAAS, to help you prepare publication quality figures: https://journals.plos.org/plosone/s/figures#loc-tools-for-figure-preparation.

NAAS will assess whether your figures meet our technical requirements by comparing each figure against our figure specifications.

# Author comment: Author response to Decision Letter 1

**DOI:** 10.1371/journal.pone.0348504.r002

10 Apr 2026

Dear Dr. Branko Aleksic, Academic Editor, PLOS ONE

We would like to thank you and the reviewers for the thoughtful and constructive comments regarding our manuscript, "Plasma vitamin C levels are associated with brain structural networks on MRI: A large cohort study" (ONE-D-26-01139). We have carefully considered each of the comments and revised the manuscript accordingly. We believe that these revisions have substantially strengthened the interpretative balance, reproducibility, and clinical relevance of our study.

Below, we provide a point-by-point response to the comments from the Academic Editor and the Reviewers. For clarity, the reviewers’ comments are in bold, followed by our responses. All page and line numbers refer to the revised manuscript with track changes.

--------------------------------------------------------------------------------

Responses to Journal Requirements

Journal Requirement #2: Overlapping text We noticed you have some minor occurrence of overlapping text with previous publications... In your revision ensure you cite all your sources (including your own works), and quote or rephrase any duplicated text outside the methods section. Response: We sincerely apologize for the overlapping text. In accordance with your instructions, we have carefully reviewed the manuscript and completely rephrased the duplicated sentences outside the Methods section. Specifically, we have thoroughly rewritten the explanation of the source-based morphometry (SBM) technique in the Introduction, as well as the descriptions of the study limitations (regarding the cross-sectional design, sample demographics, and unmeasured confounders) in the Discussion section. We have also ensured that our previous works are appropriately cited. These changes ensure the originality of the text while preserving the intended scientific meaning.

Journal Requirements #3 and #4: Competing Interests, Funding Statement, and Data Availability Response: We thank the academic editor for the guidance. As requested, we have provided the updated Funding Statement and Competing Interests Statement in our Cover Letter. These statements accurately declare the commercial affiliation, articulate the specific roles of the authors, and explicitly confirm that this commercial affiliation does not alter our adherence to PLOS ONE policies on sharing data and materials. We have also provided a detailed Data Availability Statement specifying the contact information (coi@hirosaki-u.ac.jp) for interested researchers to apply for data access.

Journal Requirement #5: Ethics Statement Response: In accordance with your instructions, we have moved the ethics statement from the Declarations section to the beginning of the Methods section (now titled "Ethics Statement"), and deleted it from the end of the manuscript.

--------------------------------------------------------------------------------

Responses to Additional Editor Comments

Comment #0-1: Causal language should be tempered given the cross-sectional design, and conclusions regarding maintenance of brain structure or DMN connectivity should be reframed as associative. The magnitude and clinical relevance of the reported effect sizes should be more explicitly discussed. Response: We sincerely appreciate your valuable comments. We have carefully revised the manuscript to temper causal language, reframing our conclusions as associative and hypothesis-generating throughout the Abstract, Discussion, and Conclusion. We have also explicitly discussed the magnitude and clinical relevance of our effect sizes in the revised Discussion. Please see our detailed responses to Reviewer #1-1 and Reviewer #1-2 below for the specific textual changes.

Comment #0-2: Additional clarification is needed regarding the statistical approach, including the rationale for using both correlation and regression analyses and confirmation that model assumptions were assessed. The identification and interpretation of DMN-related components—particularly the opposing direction observed for one posterior component—require clearer explanation. The limitations section should more explicitly acknowledge potential residual confounding and the use of a single plasma vitamin C measurement. Clarification of the clinical relevance, including the relationship with MMSE, would further strengthen the manuscript. Response: We appreciate your insightful suggestions. We have clarified the rationale for our statistical approach and the verification of regression assumptions (see Response to Reviewer #1-2a). We have provided a clearer biological interpretation of the opposing directionality of Component 135 based on its relationship with aging (see Response to Reviewer #1-3). We have also expanded the limitations section to address potential residual confounding and the single vitamin C measurement (see Responses to Reviewer #1-4 and #1-5). Finally, we have added new analyses demonstrating the relationship between these variables and cognitive performance (MMSE) to strengthen clinical relevance (see Response to Reviewer #2-4).

--------------------------------------------------------------------------------

Responses to Reviewer #1

Comment #1-1: The study is cross-sectional, yet some statements in the Discussion and Conclusion imply a causal role of vitamin C in maintaining brain structure and DMN connectivity. The authors should consistently temper their language and clearly emphasize that causal inferences cannot be made. Statements suggesting “interventions” or “maintaining brain integrity” should be reframed as hypothesis-generating rather than evidence of effect. Response: We sincerely thank the reviewer for this important feedback. We completely agree that causal inferences cannot be drawn from our cross-sectional data. We have carefully revised the manuscript to temper our language throughout the Abstract, Discussion, and Conclusion. Statements that previously implied causality or suggested clinical interventions have been reframed in associative and hypothesis-generating terms. For example, in the Abstract (Conclusions), we modified the text from: [Original] "In conclusion, plasma vitamin C levels may be essential for maintaining the structural integrity of the gray matter and for DMN connectivity." [Revised] "In conclusion, our findings demonstrate that plasma vitamin C levels are positively associated with the structural integrity of the gray matter and DMN connectivity, generating the hypothesis that vitamin C may play a role in brain health." We have also replaced words such as "predictors" with "independently associated with" throughout the text.

Comment #1-2: Although several associations reach statistical significance, the standardized β coefficients and correlation coefficients are relatively small. The authors should explicitly discuss the magnitude and potential clinical significance of these effects, rather than focusing primarily on p-values. A brief comparison with effect sizes reported in prior MRI–nutrition studies would strengthen the interpretation. Response: We appreciate this insightful comment. We have added a new paragraph to the Discussion section to explicitly discuss the magnitude and clinical relevance of our findings. Although the effect sizes in our study are relatively modest (standardized β and correlation coefficients of approximately 0.1), they are highly comparable to those reported in other large cohort studies investigating the relationship between nutrition and brain MRI markers. Furthermore, in massive epidemiological datasets (e.g., the UK Biobank), the effect sizes of established major lifestyle risk factors—including smoking and hypertension—on global brain volumes are reported to be similarly modest. Therefore, from a public health perspective, even such small associations can be clinically meaningful at the population level. Additionally, we have updated the p-values to reflect the actual robust significance levels (e.g., p < 0.001) in the revised text, replacing the previous rounded "p < 0.05" reporting.

In the revised manuscript, we have added the following paragraph to the Discussion section:

[Revised text] "Although the associations between plasma vitamin C levels, GMV, and DMN connectivity were statistically significant, the effect sizes (standardized β and correlation coefficients of approximately 0.1) were relatively modest. However, the contribution of a single nutritional or lifestyle factor to overall brain structure and large-scale networks is generally small in community-dwelling cohorts. Our effect sizes are not only comparable to those reported in other large cohort studies investigating the relationship between nutrition and brain MRI markers [1] [2], but also reach a magnitude similar to that of established major lifestyle risk factors. For instance, a recent large-scale MRI study in a healthy Japanese cohort demonstrated that the effect sizes of established vascular and metabolic risk factors on gray matter volume—such as systolic blood pressure (r = -0.10) and fasting blood glucose (r = -0.12)—are similarly modest [3]. Furthermore, in massive epidemiological datasets such as the UK Biobank (N > 9,000), the effect sizes of individual risk factors including smoking and hypertension on global brain volumes are reported to be on a similar scale (standardized β ranging from approximately 0.05 to 0.10) [4]. From a public health perspective, even such small associations can be clinically meaningful at the population level [5]. The life-long accumulation of adequate nutritional status, such as optimal vitamin C levels, may play a supportive role in preserving cognitive reserve and mitigating age-related brain atrophy and network alterations [6]. Humans cannot synthesize vitamin C; thus, it must be obtained exclusively through the dietary intake of fruits and vegetables such as citrus fruits, berries, tomatoes, potatoes, and green leafy vegetables."

Comment #1-2a: The rationale for using both Spearman correlation and multiple regression should be briefly clarified. It would be helpful to specify whether assumptions for regression analyses were formally checked. Response: We have clarified our statistical approach in the revised Methods section. In accordance with standard epidemiological modeling practices, we first used Spearman’s correlation to evaluate the unadjusted, monotonic relationships (the full correlation matrix is now provided in Supplementary Table S1). Subsequently, we employed multiple regression analysis to determine independent associations after adjusting for potential confounders. We also formally assessed the assumptions for the multiple regression models. Multicollinearity was evaluated using the Variance Inflation Factor (VIF), with all values < 3. Furthermore, the normality and homoscedasticity of the residuals were confirmed through visual inspection of diagnostic plots. These methodological details have been added to the Statistical analyses section.

In the revised manuscript, we have modified the text as follows:

[Revised text] (Methods - Statistical analyses) "Spearman’s correlation analyses were conducted separately to evaluate the unadjusted associations between plasma vitamin C levels and GMV/ICV, WMV/ICV, and each of the three DMN components. The full correlation matrix, including all clinical and demographic variables, is provided in Supplementary Table S1. Multiple regression analysis was used to investigate the independent associations between brain volume measurements (GMV/ICV ratio as an indicator of global GM atrophy and WMV/ICV ratio as an indicator of global WM atrophy), DMN connectivity, and plasma vitamin C levels after adjusting for potential confounders. Assumptions for the multiple regression models were formally assessed prior to interpretation. Multicollinearity among independent variables was evaluated using the Variance Inflation Factor (VIF); all VIF values were below 3, indicating no significant multicollinearity. Additionally, the normality and homoscedasticity of the residuals were confirmed through visual inspection of diagnostic plots."

Comment #1-3: One DMN component (posterior DMN-II) shows a negative association with plasma vitamin C levels, while others show positive associations. This opposing directionality requires clearer biological interpretation. Response: We agree that a clearer biological interpretation is necessary. We further investigated the relationships between these DMN components and age (Supplementary Table S1). We found that Components 85 and 100 exhibited negative correlations with age, representing typical structural networks that naturally decline with aging. Conversely, Component 135 showed a strong positive correlation with age, indicating an age-related structural alteration pattern that becomes more pronounced in older adults. Therefore, the negative association between plasma vitamin C and Component 135 suggests that optimal vitamin C status might effectively suppress the progression of this age-related aberrant network alteration. We have explicitly added this biological interpretation to the Discussion section.

In the revised manuscript, we have added the following paragraph to the Discussion section:

[Revised text] "Interestingly, our results demonstrated an opposing directionality among the DMN components: plasma vitamin C levels were positively associated with the anterior DMN (Component 85) and posterior DMN-I (Component 100), but negatively associated with the posterior DMN-II (Component 135). This apparent discrepancy can be biologically interpreted through their distinct relationships with aging. In our dataset, Components 85 and 100 exhibited negative correlations with age (Supplementary Table S1), representing typical structural networks that naturally decline with aging. Conversely, Component 135 showed a strong positive correlation with age, indicating an age-related structural alteration pattern that becomes more pronounced in older adults. Previous neuroimaging studies have established that DMN subsystems undergo heterogeneous and opposing changes during aging, with some networks demonstrating accelerated decline and others exhibiting relative increases or shifting connectivity patterns [7] [8]. Therefore, the positive associations with Components 85 and 100 suggest that vitamin C may help preserve normal DMN integrity, whereas the negative association with Component 135 suggests that vitamin C might effectively suppress the progression of age-related aberrant structural network changes."

Comment #1-3a: DMN components were identified by expert neuroradiologists. While acceptable, reporting inter-rater agreement or referencing a standardized atlas-based confirmation would improve reproducibility.

Response: We completely agree that providing these details improves reproducibility. In the revised Methods section (Image processing for SBM), we have clarified that two experienced board-certified neuroradiologists independently evaluated the independent components. The inter-rater agreement between the two raters was 100%. Furthermore, this visual identification was guided by cross-referencing the spatial patterns with a standardized brain functional atlas tailored for late adulthood cohorts (Atlas55+) [9].

In the revised manuscript, we have modified the text as follows:

[Revised text] (Methods - Image processing for SBM) "From the extracted independent components, two experienced neuroradiologists (S.K. with 24 years of experience, and K.W. with 12 years of experience) independently identified the DMN-related GM structural networks. This identification was performed by visually inspecting each component against established patterns documented in prior research, specifically the atlases of brain networks tailored for an elderly cohort (Atlas55+) [9]. Specifically, the anatomical criteria for identifying the DMN included the prominent inclusion of the medial prefrontal cortex and anterior cingulate cortex for the anterior DMN, and the posterior cingulate cortex, precuneus, inferior parietal cortex, and lateral temporal cortices for the posterior DMNs. The inter-rater agreement for the selection of the DMN-related components between the two neuroradiologists was 100%."

Comment #1-4: Important lifestyle and nutritional factors

Attachment Submitted filename: Response to Reviewers.docx

# Editor report: Decision Letter 1

**DOI:** 10.1371/journal.pone.0348504.r003

**Authors:** Branko Aleksic

16 Apr 2026

<p>Plasma vitamin C levels are associated with brain structural networks on MRI: A large cohort study

PONE-D-26-01139R1

Dear Dr. Shintaku,

We’re pleased to inform you that your manuscript has been judged scientifically suitable for publication and will be formally accepted for publication once it meets all outstanding technical requirements.

Within one week, you’ll receive an e-mail detailing the required amendments. When these have been addressed, you’ll receive a formal acceptance letter and your manuscript will be scheduled for publication.

An invoice will be generated when your article is formally accepted. Please note, if your institution has a publishing partnership with PLOS and your article meets the relevant criteria, all or part of your publication costs will be covered. Please make sure your user information is up-to-date by logging into Editorial Manager at Editorial Manager® and clicking the ‘Update My Information' link at the top of the page. For questions related to billing, please contact billing support.

If your institution or institutions have a press office, please notify them about your upcoming paper to help maximize its impact. If they’ll be preparing press materials, please inform our press team as soon as possible -- no later than 48 hours after receiving the formal acceptance. Your manuscript will remain under strict press embargo until 2 pm Eastern Time on the date of publication. For more information, please contact onepress@plos.org.

Kind regards,

Branko Aleksic, MD, PhD

Academic Editor

PLOS One

Additional Editor Comments (optional):

Excellent paper

Reviewers' comments:

# Editor report: Acceptance letter

**DOI:** 10.1371/journal.pone.0348504.r004

**Authors:** Branko Aleksic

PONE-D-26-01139R1

PLOS One

Dear Dr. Shintaku,

I'm pleased to inform you that your manuscript has been deemed suitable for publication in PLOS One. Congratulations! Your manuscript is now being handed over to our production team.

At this stage, our production department will prepare your paper for publication. This includes ensuring the following:

* All references, tables, and figures are properly cited

* All relevant supporting information is included in the manuscript submission,

* There are no issues that prevent the paper from being properly typeset

You will receive further instructions from the production team, including instructions on how to review your proof when it is ready. Please keep in mind that we are working through a large volume of accepted articles, so please give us a few days to review your paper and let you know the next and final steps.

Lastly, if your institution or institutions have a press office, please let them know about your upcoming paper now to help maximize its impact. If they'll be preparing press materials, please inform our press team within the next 48 hours. Your manuscript will remain under strict press embargo until 2 pm Eastern Time on the date of publication. For more information, please contact onepress@plos.org.

You will receive an invoice from PLOS for your publication fee after your manuscript has reached the completed accept phase. If you receive an email requesting payment before acceptance or for any other service, this may be a phishing scheme. Learn how to identify phishing emails and protect your accounts at https://explore.plos.org/phishing.

If we can help with anything else, please email us at customercare@plos.org.

Thank you for submitting your work to PLOS ONE and supporting open access.

Kind regards,

PLOS ONE Editorial Office Staff

on behalf of

Dr Branko Aleksic

Academic Editor

PLOS One
